# Mouse models of uveal melanoma: Strengths, weaknesses, and future directions

**DOI:** 10.1111/pcmr.12853

**Published:** 2020-01-22

**Authors:** Jackson R. Richards, Jae Hyuk Yoo, Donghan Shin, Shannon J. Odelberg

**Affiliations:** ^1^ Department of Oncological Sciences University of Utah Salt Lake City UT USA; ^2^ Program in Molecular Medicine University of Utah Salt Lake City UT USA; ^3^ Department of Internal Medicine Division of Cardiovascular Medicine University of Utah Salt Lake City UT USA; ^4^ Department of Neurobiology and Anatomy University of Utah Salt Lake City UT USA

**Keywords:** melanoma, mouse, transgenic mouse, uveal, xenograft

## Abstract

Uveal melanoma is the most common primary malignancy of the eye, and a number of discoveries in the last decade have led to a more thorough molecular characterization of this cancer. However, the prognosis remains dismal for patients with metastases, and there is an urgent need to identify treatments that are effective for this stage of disease. Animal models are important tools for preclinical studies of uveal melanoma. A variety of models exist, and they have specific advantages, disadvantages, and applications. In this review article, these differences are explored in detail, and ideas for new models that might overcome current challenges are proposed.

## 1 INTRODUCTION

Uveal melanoma is a rare (estimated incidence of 6 cases per million) and unique subtype of melanoma that arises in the uveal tract of the eye, most commonly in the choroid (Damato & Damato, 2012; McLaughlin et al., 2005). Local interventions, such as radiation therapy and enucleation, are effective at treating the primary tumor (Krantz, Dave, Komatsubara, Marr, & Carvajal, 2017). However, up to half of the patients will develop metastatic disease, predominantly to the liver (Rietschel et al., 2005). For these patients, liver‐directed therapy and participation in clinical trials are recommended, but most die from their disease, and median survival is only 10.2 months (Khoja et al., 2019; Kujala, Makitie, & Kivela, 2003; National Comprehensive Cancer Network).

Despite this, great strides have been made in understanding the molecular features of uveal melanoma. In the past decade, the collective work from several groups has led to the identification of important recurrent mutations and overactive signaling pathways in this cancer. Early oncogenic driver mutations occur in a nearly mutually exclusive pattern in the guanine nucleotide‐binding protein subunit alpha‐q/11 signaling pathway (Field et al., 2018; Moore et al., 2016; Robertson et al., 2017). This includes constitutively active variants of *GNAQ* and *GNA11*, which are found in over 90% of cases (Van Raamsdonk et al., 2009, 2010). A smaller subset of tumors harbor activating mutations in the G protein‐coupled receptor cysteinyl leukotriene receptor 2 (*CYSLTR2*) or phospholipase C beta 4 (*PLCB4*) (Johansson et al., 2016; Moore et al., 2016). There is a second node of nearly mutually exclusive mutations that classifies uveal melanomas and affects prognosis. Inactivating mutations are found in BRCA1‐associated protein 1 (*BAP1*), while recurrent point mutations are observed in the eukaryotic translation initiation factor 1A X‐linked (*EIF1AX*) or a splicing factor such as *SF3B1* (Field et al., 2018; Harbour et al., 2010, 2013; Martin et al., 2013).

The molecular makeup of a particular uveal melanoma has significant implications for predicting metastasis. Most importantly, tumors with loss‐of‐function *BAP1* mutations carry the worst prognosis, as approximately 84% of metastatic uveal melanomas are of this subtype (Harbour et al., 2010; Shain et al., 2019). Specific cytogenetic alterations have also been well described in this cancer (Aalto, Eriksson, Seregard, Larsson, & Knuutila, 2001; Anbunathan, Verstraten, Singh, Harbour, & Bowcock, 2019). Monosomy 3 co‐occurs with *BAP1* mutation, thereby eliminating both functional alleles (Field et al., 2018; Robertson et al., 2017). 6q loss, 1q gain, and 8q gain are also significantly enriched in uveal melanoma metastases (Ehlers, Worley, Onken, & Harbour, 2005; Hammond et al., 2015; Shain et al., 2019).

These discoveries were largely enabled by the analysis of patient tumor specimens and have greatly advanced our understanding of the molecular underpinnings of uveal melanoma tumorigenesis and their prognostic significance. Various animal models have likewise been indispensable in elucidating the biology and potential therapeutic vulnerabilities of this cancer (Cao & Jager, 2015; Stei, Loeffler, Holz, & Herwig, 2016; Yang, Cao, & Grossniklaus, 2015). In the past several years, there have been many promising preclinical studies that have used these models to identify novel treatment strategies, several of which are now in the early stages of clinical trials (Vivet‐Noguer, Tarin, Roman‐Roman, & Alsafadi, 2019; Yang, Manson, Marr, & Carvajal, 2018).

In this review article, we discuss the strengths and weaknesses of existing animal models of uveal melanoma, with an emphasis on mouse models. We also identify unmet needs that will require future model development and refinement. The goal of any animal model of uveal melanoma should be to faithfully recapitulate the processes of tumor initiation, growth, metastasis, and response to therapy as observed in patients with this disease.

## 2 ANIMAL MODELS OF UVEAL MELANOMA

Though the focus of this review is mouse models of uveal melanoma, other species certainly have their advantages. Rabbits (*Oryctolagus cuniculus*), for example, have large eyes that facilitate the implantation of tumor cells and subsequent monitoring using techniques such as fundoscopy, ultrasound, and magnetic resonance imaging (Bontzos & Detorakis, 2017; Gao, Tang, Liu, Yang, & Liu, 2018). The zebrafish (*Danio rerio*) is a model organism that has been used more widely in many scientific fields in recent years (Meyers, 2018). Both xenograft (Fornabaio et al., 2018; van der Ent et al., 2014) and transgenic (Mouti, Dee, Coupland, & Hurlstone, 2016; Perez, Henle, Amsterdam, Hagen, & Lees, 2018) zebrafish models of uveal melanoma have been developed. These models are excellent for high‐throughput pharmacologic screening and in vivo microscopy. The genetic models have yielded valuable insights into uveal melanoma signaling, such as the establishment of the importance of YAP activation in the initiation of this cancer. However, tumorigenesis in these models required mutation of p53, and metastasis was difficult to assess because of the induction of multiple primary tumors (Mouti et al., 2016; Perez et al., 2018).

Mice (*Mus musculus*) are the most widely used laboratory animal in the study of uveal melanoma (Cao & Jager, 2015). Their fecundity, gestation time, and size make them the most cost‐effective mammalian model (Zuberi & Lutz, 2016). Furthermore, genetic manipulation of mice has produced various strains that are used in many uveal melanoma models. The primary goals of this review are to compare the different types of mouse models of uveal melanoma and propose directions for further development.

## 3 INOCULATION SITES

**Figure 1:**
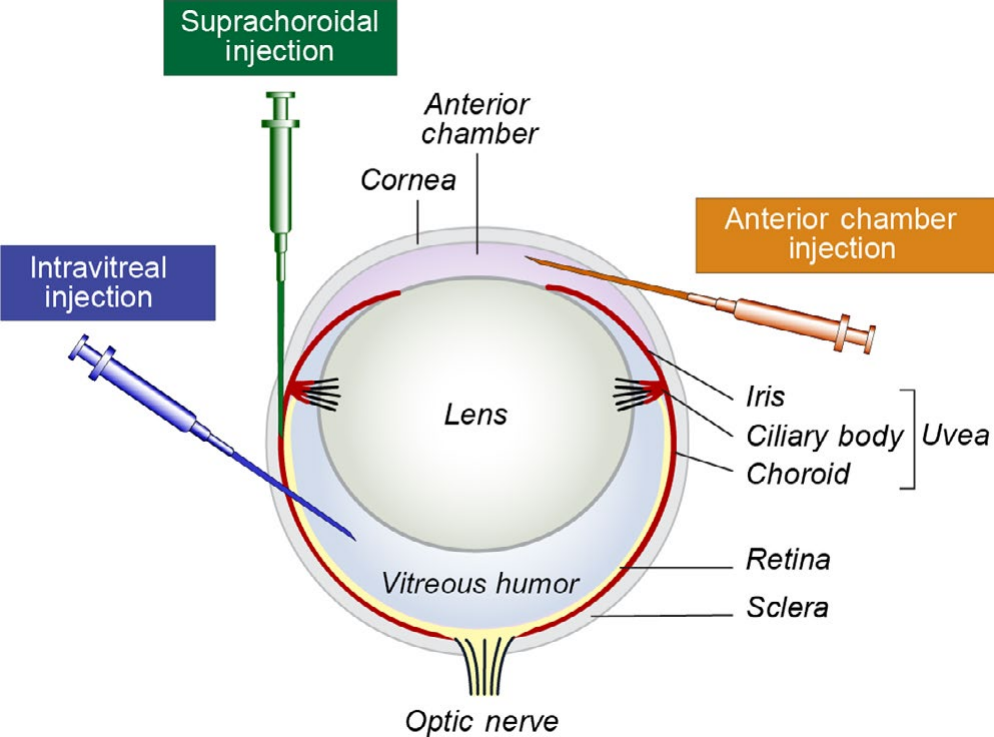
Routes of injection for orthotopic models of primary uveal melanoma. The needle trajectories for the three most commonly used types of injections are depicted (anterior chamber in orange, suprachoroidal in green, and intravitreal in blue). All three result in growth of cells in the uveal tract and therefore produce orthotopic models of uveal melanoma

The majority of murine models of uveal melanoma require the inoculation of cells or tumors into mice. Some uveal melanoma cell lines can be grown subcutaneously, which is convenient for measuring growth and response to therapy. However, others grow poorly subcutaneously but flourish in the tissue from which they were derived (Ozaki et al., 2016). In these cases, orthotopic models are preferable and may better model the human disease. Models of primary uveal melanoma in which the route of inoculation results in growth in the iris, ciliary body, or choroid are considered orthotopic (Figure 1). Inoculation of cells into the anterior chamber of the eye was one of the first techniques developed and reliably produces tumors in the iris that are capable of metastasis (Niederkorn, 1984). In 2000, a suprachoroidal injection technique was described in which cells are deposited into the posterior compartment (not to be confused with posterior chamber) of the eye (Dithmar, Rusciano, & Grossniklaus, 2000). In this approach, the needle is inserted through the limbus and into the choroid. Injected cells occupy the suprachoroidal space and likely spill into the subretinal space and vitreous. This technique is advantageous because it rapidly produces tumors in the choroid and ciliary body, the sites at which uveal melanoma most commonly occurs in patients. Furthermore, it reduces extraocular growth as compared to transconjunctival inoculations and consistently produces distant metastases (Tables 1 and 2a,b). A third type of orthotopic model is intravitreal injection. Although uveal melanoma does not arise in the vitreous humor, this environment is supportive of tumor growth and injected cells mimic human disease by invading and involving the uveal tract (Kilian et al., 2016; Yoo et al., 2016). All three of the above inoculation methods are amenable to combination with enucleation, which allows for longer follow‐up and the study of metastatic outgrowth.

**Table 1:** Syngeneic mouse cutaneous melanoma models for simulating uveal melanoma

Cell line	Source	Original publication (Laboratory of origin)	Inoculation method	Metastasis	References
B16LS9	Mouse cutaneous melanoma	Rusciano et al. (1994) (Max Burger)	Suprachoroidal	Liver, lungs, and lymph nodes	Jones et al. (2019); Dong et al. (2019); Xue et al. (2015); Yang et al. (2016), Yang and Grossniklaus (2010), Yang, Jager, and Grossniklaus (2010), Yang, Xu, Iuvone, and Grossniklaus (2006); Lattier et al. (2013); Zhang et al. (2011); Alizadeh et al. (2003); Dithmar, Rusciano, and Grossniklaus (2000), Dithmar, Rusciano, Lynn, et al. (2000); Diaz, Rusciano, Dithmar, and Grossniklaus (1999)
			Ant. chamber	Not reported	Han, Brown, and Niederkorn (2016)
			Intravitreal	Liver	Han et al. (2016); Yang et al. (2011)
			Intrasplenic		
			Intrahepatic	Not reported	Xue et al. (2015)
B16F10	Mouse cutaneous melanoma	Fidler, Gersten, and Budmen (1976) (Marilyn Budmen)	Suprachoroidal	None	Grossniklaus, Barron, and Wilson (1995)
			Ant. chamber	Not reported	el Filali et al. (2012); de Lange et al. (2012); Ly et al. (2010); Grossniklaus et al. (1995); Knisely and Niederkorn (1990);
				Lungs	Harning and Szalay (1987); Niederkorn, Sanborn, and Gamel (1987), Niederkorn (1984)
			Tail vein	Lungs	Sanborn, Niederkorn, and Gamel (1992); Niederkorn et al. (1987)
Queens	Mouse cutaneous melanoma	Harning et al. (1987) (Jeanne Szalay)	Suprachoroidal	Lungs	Rajaii et al. (2014); Grossniklaus et al. (1995)
			Ant. chamber	None	Grossniklaus et al. (1995)
				Lungs	Sanborn, Niederkorn, Kan‐Mitchell, and Albert (1992), Sanborn, Niederkorn, and Gamel (1992); Harning and Szalay (1987)
			Tail vein	Lungs	Sanborn, Niederkorn, and Gamel (1992)
HCmel12	Mouse cutaneous melanoma	Kilian et al. (2016) (Thomas Tüting)	Intravitreal	Lungs and lymph nodes	Stei, Loeffler, Kurts, et al. (2016); Kilian et al. (2016)
Oncogene‐transduced melan‐A cells	Immortalized mouse melanocyte	Bennett, Cooper, and Hart (1987) (Ian Hart)	Subcutaneous	Not reported	Moore et al. (2016)
				Lungs and liver	Van Raamsdonk et al. (2010)

Abbreviation: Ant. chamber, anterior chamber.

**Table 2:** Human uveal melanoma cell lines derived from (a) primary tumors used in mouse xenograft experiments (b) metastases used in mouse xenograft experiments

(a) Cell line (mutations)	Source	Original publication (Laboratory of origin)	Inoculation method	Metastasis	References
Mel92.1 (GNAQ^Q209L^; EIF1AX^G6D^)	Primary tumor	De Waard‐Siebinga et al. (1995) (Martine Jager)	Subcutaneous	Not reported	Faiao‐Flores et al. (2019); Forsberg et al. (2019); Kines et al. (2018); Chen et al. (2017, 2014); Ambrosini, Sawle, Musi, and Schwartz (2015); Ambrosini, Musi, Ho, Stanchina, and Schwartz, (2013); Musi, Ambrosini, Stanchina, and Schwartz, (2014); Surriga et al. (2013); Ho et al. (2012); Samadi et al. (2012); Landreville et al. (2012)
			Suprachoroidal	Liver	Dong et al. (2019)
				Not reported	Yu et al. (2014)
			Ant. chamber	Liver	Ma and Niederkorn (1998), Ma, Luyten, Luider, Jager, and Niederkorn (1996)
			Tail vein	Liver and lungs	Matatall et al. (2013)
			Intrasplenic	Liver	Barisione et al. (2015); Gangemi et al. (2014, 2012)
Mel202 (GNAQ^Q209L^; SF3B1^R625G^)	Primary tumor	Ksander, Rubsamen, Olsen, Cousins, and Streilein (1991) (J. Wayne Streilein)	Subcutaneous	Not reported	Forsberg et al. (2019)
			Ant. chamber	Liver	Ma and Niederkorn (1998), Ma, Luyten, Luider, Jager, and Niederkorn (1996)
			Intravitreal	Not reported	Yoo et al. (2016)
			Tail vein	Liver	Niederkorn, Mellon, Pidherney, Mayhew, and Anand (1993)
Mel270 (GNAQ^Q209P^)	Primary tumor	Verbik, Murray, Tran, and Ksander (1997) (Bruce Ksander)	Suprachoroidal	Not reported	Yu et al. (2014)
			Subcutaneous	Liver and lungs	Tafreshi et al. (2019)
				Lot reported	Voropaev et al. (2019); Annala et al. (2019); Kaochar et al. (2018)
			Intrasplenic	Liver	Barisione et al. (2015); Gangemi et al. (2014, 2012)
MP41 (GNA11^Q209L^)	PDX from a primary tumor	Amirouchene‐Angelozzi et al. (2014) (Sergio Roman‐Roman)	Tail vein	Liver	Faiao‐Flores et al. (2019)
T105 and T142 (GNA11^Q209L^)	Primary tumors	Mouriaux et al. (2016) (Sylvain Guérin)	Subcutaneous	Not reported	Mouriaux et al. (2016)
UMT2 (GNA11^Q209L^)	Primary tumors	Suesskind et al. (2013) (Sigrid Henke‐Fahle)	Suprachoroidal	None	Süsskind, Hurst, Rohrbach, and Schnichels (2017)

(b) Cell line	Source	Original publication (Laboratory of origin)	Inoculation site	Metastasis	References
OMM1 (GNA11^Q209L^)	Subcutis metastasis	Luyten et al. (1996) (Theo Luider)	Subcutaneous	Not reported	Zhou, Jin, Jin, Liu, and Pan (2017); Wang, Liu, Jin, Jiang, and Pan (2017); Sutmuller et al. (2000)
			Ant. chamber	Liver	Repp, Mayhew, Howard, Alizadeh, and Niederkorn (2001)
			Tail vein	Liver	Ma and Niederkorn (1995)
OMM1.3^a^ = OMM2.3 (GNAQ^Q209P^)	Liver metastasis	Verbik et al. (1997) (Bruce Ksander)	Subcutaneous	Not reported	Ambrosini et al. (2019); Jin et al. (2018); Vaqué et al. (2013)
			Suprachoroidal	Liver	Liang et al. (2012); Zhu et al. (2010)
			Retro‐orbital	Liver and lungs	Surriga et al. (2013)
			Intrasplenic	Liver	Jin et al. (2018)
TJU‐UM001 (GNAQ^Q209P^)	Liver metastasis	Yoshida et al. (2014) (Takami Sato)	Intrahepatic	Peritoneum, lymph nodes	Kageyama et al. (2017); Ozaki et al. (2016); Cheng et al. (2015)
			Intrasplenic	Liver	Piquet et al. (2019); Ozaki et al. (2016)
TJU‐UM004 (GNAQ^Q209P^)	Orbital metastasis	Cheng et al. (2015) (Takami Sato)	Intrahepatic	None	Kageyama et al. (2017)

Abbreviation: Ant. chamber: anterior chamber.

a The Mel270 cell line was derived from this patient's primary tumor. The OMM2.5 cell line (also called OMM1.5) is derived from another liver metastasis in the same patient.

The eye is bypassed in some models in order to more quickly and reliably produce large tumors in visceral organs, especially the liver. Intravenous injection into either the retro‐orbital sinus or tail vein mimics the latter part of the metastatic cascade—hematogenous dissemination, arrest and extravasation in distant sites, and metastatic colony formation and growth. The liver and lungs are the most frequently reported sites of experimental metastasis with these routes of injection (Tables 1 and 2a,b). Others have developed the intrasplenic inoculation, which consistently produces tumors in the liver (Barisione et al., 2015; Gangemi et al., 2014, 2012; Jin et al., 2018). Finally, direct implantation of cells or tumors into the liver also results in florid growth in an orthotopic model of metastatic uveal melanoma (Kageyama et al., 2017; Ozaki et al., 2016).

Irrespective of the location of injection, disease progression (e.g., tumor growth and/or metastatic dissemination) can be studied in real time using non‐invasive imaging methods such as bioluminescence imaging (Barisione et al., 2015; Surriga et al., 2013). For this technique, the injected cells have been transduced to stably express a luciferase reporter. When the graft‐bearing mice are injected with luciferin, the tumor cells emit light that can be detected by an optical imaging instrument such as Perkin Elmer's In Vivo Imaging System (IVIS). The intensity of the signal has been demonstrated to be a suitable surrogate for tumor size and thus enables dynamic evaluation of the effects of different experimental conditions on tumor progression (Cosette et al., 2016; Poeschinger, Renner, Weber, & Scheuer, 2013).

## 4 SYNGENEIC CUTANEOUS MELANOMA MOUSE MODELS FOR SIMULATING UVEAL MELANOMA

The syngeneic cutaneous melanoma mouse model has been used for decades in uveal melanoma research. In this system, cutaneous melanoma cells are implanted in mice of the same genetic background as the mice from which the line was derived. Although the cell lines used are not uveal in origin, this system allows for the investigation of intraocular growth and metastasis of melanoma cells, as many of these lines metastasize to the liver (Table 1). This mimics the behavior of uveal melanoma in humans and allows for the study of the full metastatic process, including local invasion, intravasation, survival in the blood, extravasation, and growth in distant organs. The ability to examine the interaction between tumor and host cells as the cancer progresses in an immunocompetent animal is arguably the greatest strength of this model. Additionally, recipient mice may be genetically altered in order to study specific contributions of the host in melanoma progression (Lattier, Yang, Crawford, & Grossniklaus, 2013; Stei, Loeffler, Kurts, et al., 2016).

The most widely used syngeneic model is the inoculation of C57BL/6 mice with the B16LS9 cell line, a derivative of the B16 cutaneous melanoma line that was enriched for hepatic metastatic propensity through serial in vivo passaging (Rusciano, Lorenzoni, & Burger, 1994). This cell line metastasizes to the liver from the eye, and its use has led to valuable insights into the behavior of metastatic melanoma. For instance, this model was used to show that natural killer cells and pigment‐derived epithelial factor play distinct roles in counteracting intrahepatic growth of melanoma cells (Jones, Yang, Zhang, Morales‐Tirado, & Grossniklaus, 2019). Although B16LS9 cutaneous melanoma cells were used, the histological growth patterns of the hepatic metastases in the mouse model were similar to those observed in the livers of patients with metastatic uveal melanoma (Grossniklaus et al., 2016).

The primary disadvantage of the syngeneic model is that available mouse melanoma cell lines are of cutaneous origin, so the mutations and other molecular drivers of these cells differ from those found in human uveal melanoma. Therefore, their behavior, especially their response to therapy, may differ from what is observed in patients. Interestingly, there are a few syngeneic models that do carry canonical uveal melanoma mutations. Immortalized mouse melanocytes transduced with driver mutations found in patients undergo oncogenic transformation and are capable of producing tumors and even metastases (Moore et al., 2016; Van Raamsdonk et al., 2010). Additionally, the HCmel12 mouse cutaneous melanoma cell line has been reported to carry a GNA11^Q209L^ variant (Schrage et al., 2015). Further details on other mutations in this cell line would allow for a more complete assessment of its suitability as a model for uveal melanoma. In the future, if mouse uveal melanoma cell lines could be derived from the genetically engineered mouse models discussed below, they would be powerful tools for syngeneic models. This strategy would allow for the controlled manipulation and study of bona fide uveal melanoma in an immunocompetent host.

## 5 XENOGRAFT MOUSE MODELS OF UVEAL MELANOMA

Xenograft models are another widely used approach. As the name implies, cells or tumors from a foreign source are grafted into mice. Most commonly, human uveal melanoma cell lines are used. The primary advantage of these models is that the cells are derived from patients. As such, they largely retain molecular features of the original tumor (Amirouchene‐Angelozzi et al., 2014; Griewank et al., 2012; Jager, Magner, Ksander, & Dubovy, 2016). This technique is therefore well‐suited for studying tumor signaling and response to treatment. Many recent publications detailing new potential treatments for uveal melanoma utilize xenograft models (Table 2a,b). Another advantage of xenografts is reproducibility from mouse to mouse (Gould, Junttila, & de Sauvage, 2015). Many human uveal melanoma cell lines have been described, although some are not commercially available. Frequently used cell lines with validated uveal melanoma mutations are included in Table 2a,b. Many of these xenograft models are useful for studying metastasis, as they produce tumors in organs such as the liver and lungs. It is also worth noting that some cell lines were derived from human uveal melanoma metastases. These are especially applicable for studying tumor growth in visceral organs such as the liver.

Authentication of uveal melanoma cell lines for use in xenograft models is critical. Some lines historically thought to be uveal melanoma have been found to harbor *BRAF^V600E^* mutations and are now recognized as being of cutaneous origin (Griewank et al., 2012; Yu et al., 2015). Furthermore, several of these were found by short tandem repeat (STR) analysis to be the same cell line (Folberg et al., 2008; Yu et al., 2015). Validation of uveal melanoma cell lines (including species confirmation, STR analysis, and pathogen detection) by individual laboratories is strongly encouraged. However, even after careful molecular characterization of any cancer cell line, the ability of the cells to faithfully recapitulate the behavior of their parental tumors has been questioned due to changes in molecular features that can result from culturing them in vitro (Ben‐David et al., 2018; Gillet, Varma, & Gottesman, 2013; Goodspeed, Heiser, Gray, & Costello, 2016). An example of this is that the karyotypes, including the status of chromosome 3, of several of the older cell lines differ from those of the patients’ original tumors (Jager et al., 2016). Additionally, it has been demonstrated that the gene expression profiles of uveal melanoma cell lines in culture diverge from their source tumors even after short‐term passaging (Mouriaux et al., 2016). One way to avoid these problems is to implant human tumor specimens directly into mice; this is the basis of patient‐derived xenografts.

Patient‐derived xenograft (PDX) models are relatively new in the uveal melanoma field but have demonstrated considerable translational potential. The research group led by Didier Decaudin has been the most successful and prolific in generating PDX models of uveal melanoma (Table 3). They implant fresh primary and metastatic tumor specimens in the interscapular fat pad of severe combined immunodeficient (SCID) mice and achieve an engraftment rate of 28% (Némati et al., 2010). Importantly, the tumors that grow in these mice maintain mutations, chromosomal imbalances, and histopathological features of the tumors from which they were derived (Carita, Nemati, & Decaudin, 2015). These PDX models have also been used for the derivation of new cell lines with clinically relevant features such as loss of BAP1 expression (Amirouchene‐Angelozzi et al., 2014). They have also been effective for assessing the efficacy of novel combination therapies to treat uveal melanoma (Amirouchene‐Angelozzi et al., 2016; Carita et al., 2016).

**Table 3:** Patient‐derived mouse xenograft models of uveal melanoma

PDX model	Source	Original Publication (Laboratory of origin)	Inoculation site	References
6 cases successfully grafted 3 times	Liver metastases	Kageyama et al. (2017) (Takami Sato)	Liver	Kageyama et al. (2017)
MP34, MP38, MP41, MP42, MP46, MP47, MP55, MP71, MP77, and MP80	Primary tumors	Némati et al. (2010) (Didier Decaudin)	Interscapular fat pad	Carita et al. (2016); Amirouchene‐Angelozzi et al. (2016, 2014); Némati et al. (2014, 2010); Madic et al. (2012)
MM33	Subcutis metastasis			
MM26, MM28, MM52, MM66, and MM74	Liver metastases			
ØPI‐204	Primary tumor	Heegaard, Spang‐Thomsen, and Prause (2003) (Jan Ulrik Prause)	Subcutaneous	Heegaard et al. (2003)

Another exciting recent development has been the generation of PDX models from hepatic uveal melanoma metastases (Kageyama et al., 2017). In these models, tumor specimens obtained after surgery or biopsy were surgically implanted into the livers of NOD SCID gamma mice. The authors achieved an 83% engraftment rate and found that the histology, genetics, and proteomics of the implanted tumors resembled corresponding features of patient metastases. Tumors could also be monitored by CT imaging. PDX models such as these hold promise for preclinical evaluation of experimental therapeutic compounds and the realization of personalized medicine.

Like all models, xenografts have disadvantages. The chief among these is the necessity of using immunocompromised mice. This can partially be avoided by taking advantage of the immune‐privileged nature of the anterior chamber of the eye (Niederkorn, 2012). However, this approach can only be used to study the primary tumor, and the majority of grafts spontaneously regress (Sutmuller et al., 2000). In this new era of immunotherapy, the inability to study the interplay between the tumor and host immune system, especially in sites of metastasis, is a major limitation. In uveal melanoma, this is somewhat tempered by the low response rate of patients to PD‐1 and/or CTLA4 inhibition (Algazi et al., 2016; Carvajal et al., 2017). However, other immunomodulatory pathways and cell types have been implicated in this cancer and are being actively investigated (Dougall, Kurtulus, Smyth, & Anderson, 2017; Robertson et al., 2017; Yang et al., 2016). Mice with humanized immune systems would be ideal recipients for xenograft models of all tumor types. Efforts to create such mice are ongoing but are complicated by, among other issues, graft‐versus‐host disease and interspecies differences in cytokine specificity (Allen et al., 2019; Wege, 2018). Other criticisms of xenografts, particularly PDX models, include their high cost, low engraftment rate, and low throughput (Siolas & Hannon, 2013). These are valid concerns, and the actual utility of these models in informing the treatment of patients with uveal melanoma will become more apparent in coming years.

Another approach to avoiding artifacts induced by two‐dimensional cell culturing is the use of three‐dimensional (3D) culture systems. Such “tumor organoid” models now exist for several cancers, including those arising in the colon, breast, and pancreas (Drost & Clevers, 2018; Yang, Sun, Liu, & Mao, 2018). 3D cultures derived from patient tumor specimens can be grafted into mice (patient‐derived organoid xenografts) and faithfully match the molecular phenotypes and even treatment responses of the source tumors (Sachs et al., 2018; Vlachogiannis et al., 2018). Some even allow for the study of the tumor microenvironment, as they incorporate stromal cells such as cancer‐associated fibroblasts and lymphocytes (Neal et al., 2018). In the uveal melanoma literature, there have been a few reports of 3D cultures in which cells form tumorspheres (Angi, Versluis, & Kalirai, 2015; Lapadula et al., 2019; Valyi‐Nagy et al., 2018). Further work is needed to determine the feasibility of generating such cultures from patient tumors and whether these 3D cell models better reflect the biology of their parental tumors. If so, they may serve as superior tools for both in vitro assays and xenograft models.

## 6 GENETICALLY ENGINEERED MOUSE MODELS (GEMMS) OF UVEAL MELANOMA

The third class of mouse models of uveal melanoma encompasses mice that have been genetically engineered to produce tumors. The primary advantage of these models is that they make it possible to study autochthonous tumorigenesis in an immunocompetent host. In particular, the contribution of specific genetic alterations to oncogenic signaling and disease progression can be assessed (Kersten, de Visser, van Miltenburg, & Jonkers, 2017; Zitvogel, Pitt, Daillere, Smyth, & Kroemer, 2016).

Older models include transgenic mice in which pigment cell‐specific promoters of genes such as *Tyrosinase* drive expression of the SV40 large T antigen or HRAS, although some of these tumors originate from the retinal pigment epithelium rather than the uvea (Kramer, Powell, Wilson, Salvatore, & Grossniklaus, 1998; Syed et al., 1998; Tolleson et al., 2005). In the Tg(*Grm1*) model, the *Dopachrome tautomerase* (*Dct*) promoter controls expression of the metabotropic glutamate receptor to produce both uveal melanoma and cutaneous melanoma (Schiffner et al., 2014). RET‐driven GEMMs develop melanocytic neoplasms throughout the body, including in the uveal tract (Eyles et al., 2010; Kato et al., 1998). The major weakness of all of these models is that they are driven by molecular changes not observed in patients with uveal melanoma; this limits their clinical applicability.

In the years since the discovery of *GNAQ* and *GNA11* as the main oncogenic drivers of uveal melanoma, three genetically engineered mouse models using these genes have been published (Table 4). In the first, a Tet‐on system was used to induce GNAQ^Q209L^ expression in mice deficient for p16^Ink4a^ and p19^Ink4b^ (Feng et al., 2014). Although over half of the mice developed melanocytic cutaneous lesions by 9 months, there was no report of uveal melanoma. Despite this, cutaneous tumors in this model demonstrated YAP activation downstream of oncogenic GNAQ. Another seminal paper published simultaneously reached the same conclusion and demonstrated in vivo efficacy of a YAP inhibitor using a xenograft model of uveal melanoma (Yu et al., 2014).

**Table 4:** Genetically engineered mouse models of uveal melanoma

Model genotype	Induction	Phenotype	Original Publication (Laboratory of origin)
*Dct‐rtTA*/+; *tet‐HA‐GNAQ* ^Q209L^/+; *p16p19^KO^*	5‐ to 6‐week‐old mice; doxycycline in food	>50% of mice developed cutaneous melanoma; no report of lesions in the uveal tract	Feng et al. (2014) (J. Silvio Gutkind)
*Rosa26‐floxed stop‐GNAQ* ^Q209L^/+; *Mitf‐cre/+*	Embryonic (E15.5) activation by constitutive Cre driver	Skin hyperpigmentation overt uveal melanoma and occasional dermal melanoma at 3 months in 15/15 mice; melanocytic neoplasia of the leptomeninges, harderian gland, cochlea, and vestibular system; putative metastases in the lungs at 3 months in 18/19 mice	Huang et al. (2015) (Catherine Van Raamsdonk)
*Rosa26‐floxed stop‐GNAQ* ^Q209L^/+; *Tyrosinase‐creER/+*	8‐week‐old mice; daily IP injection of tamoxifen and tail dip in 4‐HT for 5 days	Skin hyperpigmentation; melanocytic hyperplasia of the uveal tract (but not overt melanoma) in 3/3 mice	
*R26‐LSL‐GNA11* ^Q209L^/+; *Tyrosinase‐creER^T2^*/+	4‐week‐old mice; single IP injection of tamoxifen	Skin hyperpigmentation; overt uveal and dermal melanoma at 6 months in 50% of mice; melanocytic neoplasia of the leptomeninges, third ventricle, harderian gland, and heart; putative metastases in axillary lymph nodes and lungs at 3‐6 months in 100% of mice	Moore et al. (2018) (Yu Chen)
*R26‐LSL‐GNA11* ^Q209L^/+; *BAP1^lox/lox^*; *Tyrosinase‐creER^T2^*/+		Compared to above: increased dermal melanoma burden and proliferative index, no change in number or size of uveal melanoma tumors or lung lesions	
*AAV5‐CMV‐Cre or AAV5‐Trp2‐GFPCre; Lats1^f/f^; Lats2^f/f^*	2‐ to 4‐month‐old mice; suprachoroidal injection of AAV	Eye bulging at 2 months and uveal melanoma formation at 6 months in 12/14 and 8/10 mice, respectively	Li et al. (2019) (Junhao Mao)
*AAV5‐Trp2‐GFPCre; Lats1^f/f^; Lats2^f/f^; LSL‐KrasG12D*		Compared to above: larger uveal melanoma tumors in 7/7 mice and reduced survival (<4 months)	

In a different model, the expression of GNAQ^Q209L^ in a lox–stop–lox conditional knock‐in allele inserted at the *Rosa26* locus produced uveal melanoma in 3 months with 100% penetrance (Huang, Urtatiz, & Van Raamsdonk, 2015). Furthermore, it appears that cells from these tumors intravasate into blood vessels and metastasize to the lungs. Mice also developed dermal melanomas and melanocytic neoplasms at other sites, including the leptomeninges and inner ear. This model uses *Mitf‐cre* to initiate oncogene expression. Lastly, another model in which a similar conditional knock‐in allele encoding GNA11^Q209L^ is activated by the inducible *Tyrosinase‐creER^T2^* produced a comparable phenotype, albeit at a later timepoint (Moore et al., 2018). When *Bap1* deletion was combined with GNA11^Q209L^ expression, uveal melanomas were unexpectedly smaller. However, skin melanoma burden increased, as did cellular proliferation of these tumors. Comparative genomics from this model identified RasGRP3 as a critical signaling node upstream of MAPK pathway activation, a finding that had been independently reported by another group that used orthogonal methods (Chen et al., 2017).

These models have shed light on key features of uveal melanomagenesis. First, they demonstrate that *GNAQ* and *GNA11* are potent oncogenes. The deletion of tumor suppressors was not required to form uveal melanoma; indeed, in the second model, the expression of the human *GNAQ* transgene was only 3.3% of that of the murine wild‐type allele as measured by RT‐PCR of primary melanocyte cultures from the affected mice (Huang et al., 2015). Second, they illuminate differences between the pigment cell‐specific promoters used in induction. The constitutive expression of *Mitf‐cre* beginning at E15.5 likely explains the earlier onset of tumor formation in the GNAQ^Q209L^ model as compared to the GNA11^Q209L^ model in which *Tyr‐creER^T2^* is induced in 4‐week‐old mice (Huang et al., 2015; Moore et al., 2018). Interestingly, induction of *Tyr‐creER* in 8‐week‐old mice in the GNAQ^Q209L^ model did not produce overt uveal melanoma. Whether this is simply due to the differences in induction (mouse age and type of inducible Cre recombinase) or the result of differing potencies of the oncogenic drivers remains to be explored. Finally, these GEMMs illustrate which populations of melanocytes are susceptible to oncogenic transformation by these mutations and downstream activated pathways.

Like other models, these GEMMs are not without their disadvantages. Disease progression is considerably slower than in syngeneic or xenograft models due to the time required for tumor initiation. A problem specific to the GNAQ^Q209L ^model is the microphthalmia caused by the *Mitf‐cre* allele (Alizadeh, Fitch, Niswender, McKnight, & Barsh, 2008). Additionally, inserting the oncogenes in the *Rosa26* locus is somewhat artificial. A model in which an activatable allele is targeted to the endogenous mouse *Gnaq* or *Gna11* locus might better model physiologic expression of these genes. This approach has been successful in generating valuable *Braf* ^V600E^ GEMMs of cutaneous melanoma (Dankort et al., 2007; Mercer et al., 2005).

A serious obstacle encountered in the above uveal melanoma GEMMs is the induction of transgene expression in melanocytes throughout the entire body. This complicates the models in numerous ways. First, melanocytic neoplasms in other organs may cause pathology, such as the ataxic phenotype caused by melanocytosis of the vestibular system. Second, mice sometimes have to be euthanized before the ocular tumor can be fully studied because of rapid growth of melanomas arising from the dermis. Third, the study of metastasis is difficult because of the number of primary tumors, including some that develop in vital organs such as the heart (Huang et al., 2015; Moore et al., 2018).

An exciting recent publication describes a new method to overcome these issues by utilizing adeno‐associated viral delivery of Cre recombinase to the uveal tract (Li et al., 2019). In this model, the suprachoroidal injection of an AAV5‐CMV‐Cre vector produced ocular melanocytic tumors in adult mice carrying conditional null alleles of the Hippo kinases *Lats1* and *Lats2*, which normally function to suppress YAP/TAZ signaling. Furthermore, a similar vector in which Cre expression is under the control of the pigment cell‐specific *tyrosinase‐related protein 2* (*Trp2*) promoter produced a comparable phenotype. Importantly, cells of these tumors were positive for melanoma markers Melan‐A/Mart1 and HMB45 but negative for RPE65. This indicates that they arose from uveal melanocytes and not cells of the retinal pigment epithelium. Remarkably, the authors found that activation of the YAP pathway alone was both necessary and sufficient for initiation of uveal melanoma. Activation of the MAPK pathway using an inducible *Kras* ^G12D^ allele was not sufficient for tumor formation but did accelerate tumor growth and mortality in the *Lats* double knockout mice. They explored this intriguing synergy between MAPK and Hippo signaling and discovered an interactive transcriptional network in which AP1 factors amplify the oncogenic output of YAP/TEAD in uveal melanoma. The use of this AAV‐Cre system represents a significant improvement upon the aforementioned Cre driver mouse strains in that it limits oncogenic transformation to melanocytes within the uveal tract of adult mice. This is a powerful new tool that could be used in conjunction with both existing and new alleles to generate genetic mouse models that would enable the study of the entire disease process of uveal melanoma in vivo.

In addition to this AAV approach, the RCAS‐TVA system might achieve similar results. This method has been used to generate numerous GEMMs of cutaneous melanoma (Cho et al., 2015; Kircher et al., 2019; VanBrocklin, Robinson, Lastwika, Khoury, & Holmen, 2010). RCAS subgroup A is an avian retrovirus capable of infecting cells that express the TVA receptor. *Dopachrome tautomerase‐TVA* transgenic mice express this receptor in pigment‐producing cells, and this strain could be crossed with one of the conditional knock‐in alleles described above. Intraocular injection of RCAS virus that encodes for Cre would then activate oncogene expression in melanocytes of the eye. An advantage of this model over the AAV approach is targeted delivery to cells of interest such that there is no requirement for inclusion of a pigment cell‐specific promoter within the virus. This provides more room for genes of interest, which can be linked with Cre within the same viral vector to enable delivery to the same cells. Additionally, high titers of RCAS are easily produced in vitro using the chicken fibroblast DF‐1 cell line (Fisher et al., 1999), and there is no need for helper virus in these cells. Retroviruses also permit long‐term expression of genes due to genome integration, though this requires that the cells are dividing.

Despite these advancements in genetic models of uveal melanoma, the inherent differences in tumor biology between mice and humans cannot be ignored. Putative metastases that have been observed in published genetic models occur in the lungs, not the liver (Huang et al., 2015; Moore et al., 2018). Additionally, the loss of *Bap1* did not enhance the aggressiveness of uveal melanomas; in fact, the ocular phenotype was weaker, and there was no increase in size or incidence of lung lesions compared with mice expressing GNA11^Q209L^ alone (Moore et al., 2018). The basis for these differences is not understood and merits further investigation. It must also be acknowledged that the chromosomal abnormalities and epigenetic modifications observed in patients with uveal melanoma are nearly impossible to model in a mouse. Thus, while these GEMMs, as well as improved models, will continue to provide valuable insights into the progression of uveal melanoma in vivo, it is unlikely that any one model will fully recapitulate the human disease in all of its intricacies.

## 7 CONCLUSIONS

In summary, although there is no perfect mouse model of uveal melanoma, currently available models have been instrumental in elucidating critical signaling pathways and testing new therapeutic strategies for this cancer. Each type of model has distinctive strengths and weaknesses. Syngeneic models are excellent for the investigation of tumor progression in an immunocompetent host but use cutaneous melanoma cell lines. Xenograft models allow for the study of human uveal melanoma cells and tumors in a living organism, but this does not include the immune response because recipient mice must be severely immunocompromised. Genetically engineered models allow for studies of autochthonous uveal melanoma formation and dissemination, yet tumors in these mice differ from those in patients in terms of molecular complexity and metastatic behavior. Investigators should leverage the models best suited to address their specific scientific questions. Future model development should aim to overcome current limitations and further enable efforts to investigate uveal melanoma biology and develop therapies most likely to succeed in patients afflicted with this cancer.

## CONFLICT OF INTERESTS

The authors declare no conflicts of interest.

## References

[pcmr12853-bib-0001] Aalto, Y. , Eriksson, L. , Seregard, S. , Larsson, O. , & Knuutila, S. (2001). Concomitant loss of chromosome 3 and whole arm losses and gains of chromosome 1, 6, or 8 in metastasizing primary uveal melanoma. Investigative Ophthalmology & Visual Science, 42(2), 313–317. 11157859

[pcmr12853-bib-0002] Algazi, A. P. , Tsai, K. K. , Shoushtari, A. N. , Munhoz, R. R. , Eroglu, Z. , Piulats, J. M. , … Sullivan, R. J. (2016). Clinical outcomes in metastatic uveal melanoma treated with PD‐1 and PD‐L1 antibodies. Cancer, 122(21), 3344–3353. 10.1002/cncr.30258 27533448 PMC5767160

[pcmr12853-bib-0003] Alizadeh, A. , Fitch, K. R. , Niswender, C. M. , McKnight, G. S. , & Barsh, G. S. (2008). Melanocyte‐lineage expression of Cre recombinase using Mitf regulatory elements. Pigment Cell Melanoma Res, 21(1), 63–69. 18353144 10.1111/j.1755-148X.2007.00425.x

[pcmr12853-bib-0004] Alizadeh, H. , Howard, K. , Mellon, J. , Mayhew, E. , Rusciano, D. , & Niederkorn, J. Y. (2003). Reduction of liver metastasis of intraocular melanoma by interferon‐beta gene transfer. Investigative Ophthalmology & Visual Science, 44(7), 3042–3051. 12824250 10.1167/iovs.02-1147

[pcmr12853-bib-0005] Allen, T. M. , Brehm, M. A. , Bridges, S. , Ferguson, S. , Kumar, P. , Mirochnitchenko, O. , … PrabhuDas, M. (2019). Humanized immune system mouse models: Progress, challenges and opportunities. Nature Immunology, 20(7), 770–774. 10.1038/s41590-019-0416-z 31160798 PMC7265413

[pcmr12853-bib-0006] Ambrosini, G. , Do, C. , Tycko, B. , Realubit, R. B. , Karan, C. , Musi, E. , … Schwartz, G. K. (2019). Inhibition of NF‐κB‐dependent signaling enhances sensitivity and overcomes resistance to BET inhibition in uveal melanoma. Cancer Research, 79(9), 2415–2425. 10.1158/0008-5472.CAN-18-3177 30885979 PMC6643281

[pcmr12853-bib-0007] Ambrosini, G. , Musi, E. , Ho, A. L. , de Stanchina, E. , & Schwartz, G. K. (2013). Inhibition of mutant GNAQ signaling in uveal melanoma induces AMPK‐dependent autophagic cell death. Molecular Cancer Therapeutics, 12(5), 768–776. 10.1158/1535-7163.MCT-12-1020 23443802

[pcmr12853-bib-0008] Ambrosini, G. , Sawle, A. D. , Musi, E. , & Schwartz, G. K. (2015). BRD4‐targeted therapy induces Myc‐independent cytotoxicity in Gnaq/11‐mutatant uveal melanoma cells. Oncotarget, 6(32), 33397–33409. 10.18632/oncotarget.5179 26397223 PMC4741774

[pcmr12853-bib-0009] Amirouchene‐Angelozzi, N. , Frisch‐Dit‐Leitz, E. , Carita, G. , Dahmani, A. , Raymondie, C. , Liot, G. , … Schoumacher, M. (2016). The mTOR inhibitor Everolimus synergizes with the PI3K inhibitor GDC0941 to enhance anti‐tumor efficacy in uveal melanoma. Oncotarget, 7(17), 23633–23646. 10.18632/oncotarget.8054 26988753 PMC5029652

[pcmr12853-bib-0010] Amirouchene‐Angelozzi, N. , Nemati, F. , Gentien, D. , Nicolas, A. , Dumont, A. , Carita, G. , … Roman‐Roman, S. (2014). Establishment of novel cell lines recapitulating the genetic landscape of uveal melanoma and preclinical validation of mTOR as a therapeutic target. Molecular Oncology, 8(8), 1508–1520. 10.1016/j.molonc.2014.06.004 24994677 PMC5528590

[pcmr12853-bib-0011] Anbunathan, H. , Verstraten, R. , Singh, A. D. , Harbour, J. W. , & Bowcock, A. M. (2019). Integrative copy number analysis of uveal melanoma reveals novel candidate genes involved in tumorigenesis including a tumor suppressor role for PHF10/BAF45a. Clinical Cancer Research, 25(16), 5156–5166. 31227497 10.1158/1078-0432.CCR-18-3052 PMC6697622

[pcmr12853-bib-0012] Angi, M. , Versluis, M. , & Kalirai, H. (2015). Culturing uveal melanoma cells. Ocular Oncology and Pathology, 1(3), 126–132. 10.1159/000370150 27171555 PMC4847689

[pcmr12853-bib-0013] Annala, S. , Feng, X. , Shridhar, N. , Eryilmaz, F. , Patt, J. , Yang, J. , … Kostenis, E. (2019). Direct targeting of Gαq and Gα11 oncoproteins in cancer cells. Science Signalling, 12(573), eaau5948. 10.1126/scisignal.aau5948 30890659

[pcmr12853-bib-0014] Barisione, G. , Fabbi, M. , Gino, A. , Queirolo, P. , Orgiano, L. , Spano, L. , … Gangemi, R. (2015). Potential role of soluble c‐Met as a new candidate biomarker of metastatic uveal melanoma. JAMA Ophthalmology, 133(9), 1013–1021. 10.1001/jamaophthalmol.2015.1766 26068448

[pcmr12853-bib-0015] Ben‐David, U. , Siranosian, B. , Ha, G. , Tang, H. , Oren, Y. , Hinohara, K. , … Golub, T. R. (2018). Genetic and transcriptional evolution alters cancer cell line drug response. Nature, 560(7718), 325–330. 30089904 10.1038/s41586-018-0409-3 PMC6522222

[pcmr12853-bib-0016] Bennett, D. C. , Cooper, P. J. , & Hart, I. R. (1987). A line of non‐tumorigenic mouse melanocytes, syngeneic with the B16 melanoma and requiring a tumour promoter for growth. International Journal of Cancer, 39(3), 414–418. 10.1002/ijc.2910390324 3102392

[pcmr12853-bib-0017] Bontzos, G. , & Detorakis, E. T. (2017). Animal models of uveal melanoma for localized interventions. Critical Reviews in Oncogenesis, 22(3–4), 187–194. 10.1615/CritRevOncog.2018024510 29604898

[pcmr12853-bib-0018] Cao, J. , & Jager, M. J. (2015). Animal eye models for uveal melanoma. Ocular Oncology and Pathology, 1(3), 141–150. 10.1159/000370152 27172424 PMC4847684

[pcmr12853-bib-0019] Carita, G. , Frisch‐Dit‐Leitz, E. , Dahmani, A. , Raymondie, C. , Cassoux, N. , Piperno‐Neumann, S. , … Decaudin, D. (2016). Dual inhibition of protein kinase C and p53‐MDM2 or PKC and mTORC1 are novel efficient therapeutic approaches for uveal melanoma. Oncotarget, 7(23), 33542–33556. 10.18632/oncotarget.9552 27507190 PMC5085101

[pcmr12853-bib-0020] Carita, G. , Nemati, F. , & Decaudin, D. (2015). Uveal melanoma patient‐derived xenografts. Ocular Oncology and Pathology, 1(3), 161–169. 10.1159/000370154 27172261 PMC4847660

[pcmr12853-bib-0021] Carvajal, R. D. , Schwartz, G. K. , Tezel, T. , Marr, B. , Francis, J. H. , & Nathan, P. D. (2017). Metastatic disease from uveal melanoma: Treatment options and future prospects. British Journal of Ophthalmology, 101(1), 38–44. 10.1136/bjophthalmol-2016-309034 27574175 PMC5256122

[pcmr12853-bib-0022] Chen, X. U. , Wu, Q. , Depeille, P. , Chen, P. , Thornton, S. , Kalirai, H. , … Bastian, B. C. (2017). RasGRP3 mediates MAPK pathway activation in GNAQ mutant uveal melanoma. Cancer Cell, 31(5), 685–696 e686. 10.1016/j.ccell.2017.04.002 28486107 PMC5499527

[pcmr12853-bib-0023] Chen, X. , Wu, Q. , Tan, L. , Porter, D. , Jager, M. J. , Emery, C. , & Bastian, B. C. (2014). Combined PKC and MEK inhibition in uveal melanoma with GNAQ and GNA11 mutations. Oncogene, 33(39), 4724–4734. 10.1038/onc.2013.418 24141786 PMC4524511

[pcmr12853-bib-0024] Cheng, H. , Terai, M. , Kageyama, K. , Ozaki, S. , McCue, P. A. , Sato, T. , & Aplin, A. E. (2015). Paracrine effect of NRG1 and HGF drives resistance to MEK inhibitors in metastatic uveal melanoma. Cancer Research, 75(13), 2737–2748. 10.1158/0008-5472.CAN-15-0370 25952648 PMC4490069

[pcmr12853-bib-0025] Cho, J. H. , Robinson, J. P. , Arave, R. A. , Burnett, W. J. , Kircher, D. A. , Chen, G. , … Holmen, S. L. (2015). AKT1 activation promotes development of melanoma metastases. Cell Reports, 13(5), 898–905. 10.1016/j.celrep.2015.09.057 26565903 PMC4646731

[pcmr12853-bib-0026] Cosette, J. , Ben Abdelwahed, R. , Donnou‐Triffault, S. , Sautes‐Fridman, C. , Flaud, P. , & Fisson, S. (2016). Bioluminescence‐based tumor quantification method for monitoring tumor progression and treatment effects in mouse lymphoma models. Journal of Visualized Experiments, (113), e53609 10.3791/53609 PMC4993383 27501019

[pcmr12853-bib-0027] Damato, E. M. , & Damato, B. E. (2012). Detection and time to treatment of uveal melanoma in the United Kingdom: An evaluation of 2,384 patients. Ophthalmology, 119(8), 1582–1589. 10.1016/j.ophtha.2012.01.048 22503229

[pcmr12853-bib-0028] Dankort, D. , Filenova, E. , Collado, M. , Serrano, M. , Jones, K. , & McMahon, M. (2007). A new mouse model to explore the initiation, progression, and therapy of BRAFV600E‐induced lung tumors. Genes & Development, 21(4), 379–384. 10.1101/gad.1516407 17299132 PMC1804325

[pcmr12853-bib-0029] de Lange, J. , Ly, L. V. , Lodder, K. , Verlaan‐de Vries, M. , Teunisse, A. F. , Jager, M. J. , & Jochemsen, A. G. (2012). Synergistic growth inhibition based on small‐molecule p53 activation as treatment for intraocular melanoma. Oncogene, 31(9), 1105–1116. 10.1038/onc.2011.309 21765463

[pcmr12853-bib-0030] De Waard‐Siebinga, I. , Blom, D.‐J. , Griffioen, M. , Schrier, P. I. , Hoogendoorn, E. D. , Beverstock, G. , … Jager, M. J. (1995). Establishment and characterization of an uveal‐melanoma cell line. International Journal of Cancer, 62(2), 155–161. 10.1002/ijc.2910620208 7622289

[pcmr12853-bib-0031] Diaz, C. E. , Rusciano, D. , Dithmar, S. , & Grossniklaus, H. E. (1999). B16LS9 melanoma cells spread to the liver from the murine ocular posterior compartment (PC). Current Eye Research, 18(2), 125–129. 10.1076/ceyr.18.2.125.5380 10223656

[pcmr12853-bib-0032] Dithmar, S. , Rusciano, D. , & Grossniklaus, H. E. (2000). A new technique for implantation of tissue culture melanoma cells in a murine model of metastatic ocular melanoma. Melanoma Research, 10(1), 2–8. 10.1097/00008390-200002000-00001 10711634

[pcmr12853-bib-0033] Dithmar, S. , Rusciano, D. , Lynn, M. J. , Lawson, D. H. , Armstrong, C. A. , & Grossniklaus, H. E. (2000). Neoadjuvant interferon alfa‐2b treatment in a murine model for metastatic ocular melanoma: A preliminary study. Archives of Ophthalmology, 118(8), 1085–1089. 10922203 10.1001/archopht.118.8.1085

[pcmr12853-bib-0034] Dong, L. , You, S. , Zhang, Q. , Osuka, S. , Devi, N. S. , Kaluz, S. , … Van Meir, E. G. (2019). Arylsulfonamide 64B inhibits hypoxia/HIF‐induced expression of c‐Met and CXCR4 and reduces primary tumor growth and metastasis of uveal melanoma. Clinical Cancer Research, 25(7), 2206–2218. 10.1158/1078-0432.CCR-18-1368 30563937 PMC6445693

[pcmr12853-bib-0035] Dougall, W. C. , Kurtulus, S. , Smyth, M. J. , & Anderson, A. C. (2017). TIGIT and CD96: New checkpoint receptor targets for cancer immunotherapy. Immunological Reviews, 276(1), 112–120. 10.1111/imr.12518 28258695

[pcmr12853-bib-0036] Drost, J. , & Clevers, H. (2018). Organoids in cancer research. Nature Reviews Cancer, 18(7), 407–418. 10.1038/s41568-018-0007-6 29692415

[pcmr12853-bib-0037] Ehlers, J. P. , Worley, L. , Onken, M. D. , & Harbour, J. W. (2005). DDEF1 is located in an amplified region of chromosome 8q and is overexpressed in uveal melanoma. Clinical Cancer Research, 11(10), 3609–3613. 10.1158/1078-0432.CCR-04-1941 15897555

[pcmr12853-bib-0038] el Filali, M. , Ly, L. V. , Luyten, G. P. , Versluis, M. , Grossniklaus, H. E. , van der Velden, P. A. , & Jager, M. J. (2012). Bevacizumab and intraocular tumors: An intriguing paradox. Molecular Vision, 18, 2454–2467. 23077404 PMC3472924

[pcmr12853-bib-0039] Eyles, J. O. , Puaux, A.‐L. , Wang, X. , Toh, B. , Prakash, C. , Hong, M. , … Abastado, J.‐P. (2010). Tumor cells disseminate early, but immunosurveillance limits metastatic outgrowth, in a mouse model of melanoma. Journal of Clinical Investigation, 120(6), 2030–2039. 10.1172/JCI42002 20501944 PMC2877955

[pcmr12853-bib-0040] Faiao‐Flores, F. , Emmons, M. F. , Durante, M. A. , Kinose, F. , Saha, B. , Fang, B. , … Smalley, K. S. M. (2019). HDAC inhibition enhances the in vivo efficacy of MEK inhibitor therapy in uveal melanoma. Clinical Cancer Research, 25(18), 5686–5701. 31227503 10.1158/1078-0432.CCR-18-3382 PMC6744978

[pcmr12853-bib-0041] Feng, X. , Degese, M. S. , Iglesias‐Bartolome, R. , Vaque, J. P. , Molinolo, A. A. , Rodrigues, M. , … Gutkind, J. S. (2014). Hippo‐independent activation of YAP by the GNAQ uveal melanoma oncogene through a trio‐regulated rho GTPase signaling circuitry. Cancer Cell, 25(6), 831–845. 10.1016/j.ccr.2014.04.016 24882515 PMC4074519

[pcmr12853-bib-0042] Fidler, I. J. , Gersten, D. M. , & Budmen, M. B. (1976). Characterization in vivo and in vitro of tumor cells selected for resistance to syngeneic lymphocyte‐mediated cytotoxicity. Cancer Research, 36(9 pt.1), 3160–3165. 975082

[pcmr12853-bib-0043] Field, M. G. , Durante, M. A. , Anbunathan, H. , Cai, L. Z. , Decatur, C. L. , Bowcock, A. M. , … Harbour, J. W. (2018). Punctuated evolution of canonical genomic aberrations in uveal melanoma. Nature Communications, 9(1), 116 10.1038/s41467-017-02428-w PMC5760704 29317634

[pcmr12853-bib-0044] Fisher, G. H. , Orsulic, S. , Holland, E. , Hively, W. P. , Li, Y. I. , Lewis, B. C. , … Varmus, H. E. (1999). Development of a flexible and specific gene delivery system for production of murine tumor models. Oncogene, 18(38), 5253–5260. 10.1038/sj.onc.1203087 10498877

[pcmr12853-bib-0045] Folberg, R. , Kadkol, S. S. , Frenkel, S. , Valyi‐Nagy, K. , Jager, M. J. , Pe'er, J. , & Maniotis, A. J. (2008). Authenticating cell lines in ophthalmic research laboratories. Investigative Ophthalmology & Visual Science, 49(11), 4697–4701. 10.1167/iovs.08-2324 18689700 PMC2576485

[pcmr12853-bib-0046] Fornabaio, G. , Barnhill, R. L. , Lugassy, C. , Bentolila, L. A. , Cassoux, N. , Roman‐Roman, S. , … Del Bene, F. (2018). Angiotropism and extravascular migratory metastasis in cutaneous and uveal melanoma progression in a zebrafish model. Scientific Reports, 8(1), 10448 10.1038/s41598-018-28515-6 29992995 PMC6041265

[pcmr12853-bib-0047] Forsberg, E. M. V. , Lindberg, M. F. , Jespersen, H. , Alsén, S. , Bagge, R. O. , Donia, M. , … Nilsson, J. A. (2019). HER2 CAR‐T cells eradicate uveal melanoma and T‐cell therapy‐resistant human melanoma in IL2 Transgenic NOD/SCID IL2 receptor knockout mice. Cancer Research, 79(5), 899–904. 10.1158/0008-5472.CAN-18-3158 30622115

[pcmr12853-bib-0048] Gangemi, R. , Amaro, A. , Gino, A. , Barisione, G. , Fabbi, M. , Pfeffer, U. , … Ferrini, S. (2014). ADAM10 correlates with uveal melanoma metastasis and promotes in vitro invasion. Pigment Cell & Melanoma Research, 27(6), 1138–1148. 10.1111/pcmr.12306 25124714

[pcmr12853-bib-0049] Gangemi, R. , Mirisola, V. , Barisione, G. , Fabbi, M. , Brizzolara, A. , Lanza, F. , … Ferrini, S. (2012). Mda‐9/syntenin is expressed in uveal melanoma and correlates with metastatic progression. PLoS ONE, 7(1), e29989 10.1371/journal.pone.0029989 22267972 PMC3258266

[pcmr12853-bib-0050] Gao, M. , Tang, J. , Liu, K. , Yang, M. , & Liu, H. (2018). Quantitative evaluation of vascular microcirculation using contrast‐enhanced ultrasound imaging in rabbit models of choroidal melanoma. Investigative Ophthalmology & Visual Science, 59(3), 1251–1262. 10.1167/iovs.17-22197 29625446

[pcmr12853-bib-0051] Gillet, J. P. , Varma, S. , & Gottesman, M. M. (2013). The clinical relevance of cancer cell lines. Journal of the National Cancer Institute, 105(7), 452–458. 10.1093/jnci/djt007 23434901 PMC3691946

[pcmr12853-bib-0052] Goodspeed, A. , Heiser, L. M. , Gray, J. W. , & Costello, J. C. (2016). Tumor‐derived cell lines as molecular models of cancer pharmacogenomics. Molecular Cancer Research, 14(1), 3–13. 10.1158/1541-7786.MCR-15-0189 26248648 PMC4828339

[pcmr12853-bib-0053] Gould, S. E. , Junttila, M. R. , & de Sauvage, F. J. (2015). Translational value of mouse models in oncology drug development. Nature Medicine, 21(5), 431–439. 10.1038/nm.3853 25951530

[pcmr12853-bib-0055] Griewank, K. G. , Yu, X. , Khalili, J. , Sozen, M. M. , Stempke‐Hale, K. , Bernatchez, C. , … Woodman, S. E. (2012). Genetic and molecular characterization of uveal melanoma cell lines. Pigment Cell Melanoma Res, 25(2), 182–187. 10.1111/j.1755-148X.2012.00971.x 22236444 PMC3288394

[pcmr12853-bib-0056] Grossniklaus, H. E. , Barron, B. C. , & Wilson, M. W. (1995). Murine model of anterior and posterior ocular melanoma. Current Eye Research, 14(5), 399–404. 10.3109/02713689508999938 7648866

[pcmr12853-bib-0057] Grossniklaus, H. E. , Zhang, Q. , You, S. , McCarthy, C. , Heegaard, S. , & Coupland, S. E. (2016). Metastatic ocular melanoma to the liver exhibits infiltrative and nodular growth patterns. Human Pathology, 57, 165–175. 10.1016/j.humpath.2016.07.012 27476775 PMC5547398

[pcmr12853-bib-0058] Hammond, D. W. , Al‐Shammari, N. S. , Danson, S. , Jacques, R. , Rennie, I. G. , & Sisley, K. (2015). High‐resolution array CGH analysis identifies regional deletions and amplifications of chromosome 8 in uveal melanoma. Investigative Ophthalmology & Visual Science, 56(6), 3460–3466. 10.1167/iovs.14-16215 26030101

[pcmr12853-bib-0059] Han, Z. , Brown, J. R. , & Niederkorn, J. Y. (2016). Growth and metastasis of intraocular tumors in aged mice. Investigative Ophthalmology & Visual Science, 57(6), 2366–2376. 10.1167/iovs.16-19156 27138736 PMC4857834

[pcmr12853-bib-0060] Harbour, J. W. , Onken, M. D. , Roberson, E. D. , Duan, S. , Cao, L. , Worley, L. A. , … Bowcock, A. M. (2010). Frequent mutation of BAP1 in metastasizing uveal melanomas. Science, 330(6009), 1410–1413. 21051595 10.1126/science.1194472 PMC3087380

[pcmr12853-bib-0061] Harbour, J. W. , Roberson, E. D. , Anbunathan, H. , Onken, M. D. , Worley, L. A. , & Bowcock, A. M. (2013). Recurrent mutations at codon 625 of the splicing factor SF3B1 in uveal melanoma. Nature Genetics, 45(2), 133–135. 10.1038/ng.2523 23313955 PMC3789378

[pcmr12853-bib-0062] Harning, R. , & Szalay, J. (1987). Ocular metastasis of in vivo and in vitro derived syngeneic murine melanoma. Investigative Ophthalmology & Visual Science, 28(9), 1599–1604. 3623843

[pcmr12853-bib-0063] Heegaard, S. , Spang‐Thomsen, M. , & Prause, J. U. (2003). Establishment and characterization of human uveal malignant melanoma xenografts in nude mice. Melanoma Research, 13(3), 247–251. 10.1097/00008390-200306000-00004 12777978

[pcmr12853-bib-0064] Ho, A. L. , Musi, E. , Ambrosini, G. , Nair, J. S. , Deraje Vasudeva, S. , de Stanchina, E. , & Schwartz, G. K. (2012). Impact of combined mTOR and MEK inhibition in uveal melanoma is driven by tumor genotype. PLoS ONE, 7(7), e40439 10.1371/journal.pone.0040439 22808163 PMC3393714

[pcmr12853-bib-0065] Huang, J. L. , Urtatiz, O. , & Van Raamsdonk, C. D. (2015). Oncogenic G protein GNAQ induces uveal melanoma and intravasation in mice. Cancer Research, 75(16), 3384–3397. 10.1158/0008-5472.CAN-14-3229 26113083

[pcmr12853-bib-0066] Jager, M. J. , Magner, J. A. , Ksander, B. R. , & Dubovy, S. R. (2016). Uveal melanoma cell lines: Where do they come from? (An American Ophthalmological Society Thesis). Transactions of the American Ophthalmological Society, 114, T5. 28018010 PMC5161001

[pcmr12853-bib-0067] Jin, Y. , Zhang, P. , Wang, Y. , Jin, B. , Zhou, J. , Zhang, J. , & Pan, J. (2018). Neddylation blockade diminishes hepatic metastasis by dampening cancer stem‐like cells and angiogenesis in uveal melanoma. Clinical Cancer Research, 24(15), 3741–3754. 10.1158/1078-0432.CCR-17-1703 29233905

[pcmr12853-bib-0068] Johansson, P. , Aoude, L. G. , Wadt, K. , Glasson, W. J. , Warrier, S. K. , Hewitt, A. W. , … Hayward, N. K. (2016). Deep sequencing of uveal melanoma identifies a recurrent mutation in PLCB4. Oncotarget, 7(4), 4624–4631. 26683228 10.18632/oncotarget.6614 PMC4826231

[pcmr12853-bib-0069] Jones, N. M. , Yang, H. , Zhang, Q. , Morales‐Tirado, V. M. , & Grossniklaus, H. E. (2019). Natural killer cells and pigment epithelial‐derived factor control the infiltrative and nodular growth of hepatic metastases in an Orthotopic murine model of ocular melanoma. BMC Cancer, 19(1), 484 10.1186/s12885-019-5712-3 31117965 PMC6532210

[pcmr12853-bib-0070] Kageyama, K. , Ohara, M. , Saito, K. , Ozaki, S. , Terai, M. , Mastrangelo, M. J. , … Sato, T. (2017). Establishment of an orthotopic patient‐derived xenograft mouse model using uveal melanoma hepatic metastasis. Journal of Translational Medicine, 15(1), 145 10.1186/s12967-017-1247-z 28645290 PMC5481921

[pcmr12853-bib-0071] Kaochar, S. , Dong, J. , Torres, M. , Rajapakshe, K. , Nikolos, F. , Davis, C. M. , … Poulaki, V. (2018). ICG‐001 exerts potent anticancer activity against uveal melanoma cells. Investigative Opthalmology & Visual Science, 59(1), 132–143. 10.1167/iovs.17-22454 PMC5769500 29332125

[pcmr12853-bib-0072] Kato, M. , Takahashi, M. , Akhand, A. A. , Liu, W. , Dai, Y. , Shimizu, S. , … Nakashima, I. (1998). Transgenic mouse model for skin malignant melanoma. Oncogene, 17(14), 1885–1888. 10.1038/sj.onc.1202077 9778055

[pcmr12853-bib-0073] Kersten, K. , de Visser, K. E. , van Miltenburg, M. H. , & Jonkers, J. (2017). Genetically engineered mouse models in oncology research and cancer medicine. EMBO Molecular Medicine, 9(2), 137–153. 10.15252/emmm.201606857 28028012 PMC5286388

[pcmr12853-bib-0074] Khoja, L. , Atenafu, E. G. , Suciu, S. , Leyvraz, S. , Sato, T. , Marshall, E. , … Joshua, A. M. (2019). Meta‐analysis in metastatic uveal melanoma to determine progression‐free and overall survival benchmarks: An International Rare Cancers Initiative (IRCI) ocular melanoma study. Annals of Oncology, 30(8), 1370–1380. 10.1093/annonc/mdz176 31150059

[pcmr12853-bib-0075] Kilian, M. M. , Loeffler, K. U. , Pfarrer, C. , Holz, F. G. , Kurts, C. , & Herwig, M. C. (2016). Intravitreally injected HCmel12 melanoma cells serve as a mouse model of tumor biology of intraocular melanoma. Current Eye Research, 41(1), 121–128. 10.3109/02713683.2015.1004721 25658144

[pcmr12853-bib-0076] Kines, R. C. , Varsavsky, I. , Choudhary, S. , Bhattacharya, D. , Spring, S. , McLaughlin, R. , … Schiller, J. T. (2018). An infrared dye‐conjugated virus‐like particle for the treatment of primary uveal melanoma. Molecular Cancer Therapeutics, 17(2), 565–574. 10.1158/1535-7163.MCT-17-0953 29242243 PMC8063570

[pcmr12853-bib-0077] Kircher, D. A. , Trombetti, K. A. , Silvis, M. R. , Parkman, G. L. , Fischer, G. M. , Angel, S. N. , … Holmen, S. L. (2019). AKT1(E17K) activates focal adhesion kinase and promotes melanoma brain metastasis. Molecular Cancer Research, 17(9), 1787–1800. 31138602 10.1158/1541-7786.MCR-18-1372 PMC6726552

[pcmr12853-bib-0078] Knisely, T. L. , & Niederkorn, J. Y. (1990). Immunologic evaluation of spontaneous regression of an intraocular murine melanoma. Investigative Ophthalmology & Visual Science, 31(2), 247–257. 2154414

[pcmr12853-bib-0079] Kramer, T. R. , Powell, M. B. , Wilson, M. M. , Salvatore, J. , & Grossniklaus, H. E. (1998). Pigmented uveal tumours in a transgenic mouse model. British Journal of Ophthalmology, 82(8), 953–960. 10.1136/bjo.82.8.953 9828784 PMC1722710

[pcmr12853-bib-0080] Krantz, B. A. , Dave, N. , Komatsubara, K. M. , Marr, B. P. , & Carvajal, R. D. (2017). Uveal melanoma: Epidemiology, etiology, and treatment of primary disease. Clin Ophthalmol, 11, 279–289. 28203054 10.2147/OPTH.S89591 PMC5298817

[pcmr12853-bib-0081] Ksander, B. R. , Rubsamen, P. E. , Olsen, K. R. , Cousins, S. W. , & Streilein, J. W. (1991). Studies of tumor‐infiltrating lymphocytes from a human choroidal melanoma. Investigative Ophthalmology & Visual Science, 32(13), 3198–3208. 1748551

[pcmr12853-bib-0082] Kujala, E. , Makitie, T. , & Kivela, T. (2003). Very long‐term prognosis of patients with malignant uveal melanoma. Investigative Ophthalmology & Visual Science, 44(11), 4651–4659. 10.1167/iovs.03-0538 14578381

[pcmr12853-bib-0083] Landreville, S. , Agapova, O. A. , Matatall, K. A. , Kneass, Z. T. , Onken, M. D. , Lee, R. S. , … Harbour, J. W. (2012). Histone deacetylase inhibitors induce growth arrest and differentiation in uveal melanoma. Clinical Cancer Research, 18(2), 408–416. 10.1158/1078-0432.CCR-11-0946 22038994 PMC3261307

[pcmr12853-bib-0084] Lapadula, D. , Farias, E. , Randolph, C. E. , Purwin, T. J. , McGrath, D. , Charpentier, T. H. , … Benovic, J. L. (2019). Effects of oncogenic Gαq and Gα11 inhibition by FR900359 in uveal melanoma. Molecular Cancer Research, 17(4), 963–973. 30567972 10.1158/1541-7786.MCR-18-0574 PMC6445713

[pcmr12853-bib-0085] Lattier, J. M. , Yang, H. , Crawford, S. , & Grossniklaus, H. E. (2013). Host pigment epithelium‐derived factor (PEDF) prevents progression of liver metastasis in a mouse model of uveal melanoma. Clinical & Experimental Metastasis, 30(8), 969–976. 10.1007/s10585-013-9596-3 23793989 PMC3844008

[pcmr12853-bib-0086] Li, H. , Li, Q. I. , Dang, K. , Ma, S. , Cotton, J. L. , Yang, S. , … Mao, J. (2019). YAP/TAZ activation drives uveal melanoma initiation and progression. Cell Reports, 29(10), 3200–3211 e3204. 10.1016/j.celrep.2019.03.021 31801083 PMC7871510

[pcmr12853-bib-0087] Liang, Z. , Zhan, W. , Zhu, A. , Yoon, Y. , Lin, S. , Sasaki, M. , … Shim, H. (2012). Development of a unique small molecule modulator of CXCR4. PLoS ONE, 7(4), e34038 10.1371/journal.pone.0034038 22485156 PMC3317778

[pcmr12853-bib-0088] Luyten, G. P. M. , Naus, N. C. , Mooy, C. M. , Hagemeijer, A. , Kan‐Mitchell, J. , Van Drunen, E. , … Luider, T. M. (1996). Establishment and characterization of primary and metastatic uveal melanoma cell lines. International Journal of Cancer, 66(3), 380–387. 10.1002/(SICI)1097-0215(19960503)66:3<380:AID-IJC19>3.0.CO;2-F 8621261

[pcmr12853-bib-0089] Ly, L. V. , Baghat, A. , Versluis, M. , Jordanova, E. S. , Luyten, G. P. M. , van Rooijen, N. , … Jager, M. J. (2010). In aged mice, outgrowth of intraocular melanoma depends on proangiogenic M2‐type macrophages. The Journal of Immunology, 185(6), 3481–3488. 10.4049/jimmunol.0903479 20713886

[pcmr12853-bib-0090] Ma, D. , Luyten, G. P. , Luider, T. M. , Jager, M. J. , & Niederkorn, J. Y. (1996). Association between NM23‐H1 gene expression and metastasis of human uveal melanoma in an animal model. Investigative Ophthalmology & Visual Science, 37(11), 2293–2301. 8843913

[pcmr12853-bib-0091] Ma, D. , & Niederkorn, J. Y. (1995). Transforming growth factor‐beta down‐regulates major histocompatibility complex class I antigen expression and increases the susceptibility of uveal melanoma cells to natural killer cell‐mediated cytolysis. Immunology, 86(2), 263–269. 7490128 PMC1384005

[pcmr12853-bib-0092] Ma, D. , & Niederkorn, J. Y. (1998). Role of epidermal growth factor receptor in the metastasis of intraocular melanomas. Investigative Ophthalmology & Visual Science, 39(7), 1067–1075. 9620065

[pcmr12853-bib-0093] Madic, J. , Piperno‐Neumann, S. , Servois, V. , Rampanou, A. , Milder, M. , Trouiller, B. , … Stern, M.‐H. (2012). Pyrophosphorolysis‐activated polymerization detects circulating tumor DNA in metastatic uveal melanoma. Clinical Cancer Research, 18(14), 3934–3941. 10.1158/1078-0432.CCR-12-0309 22645051

[pcmr12853-bib-0094] Martin, M. , Maßhöfer, L. , Temming, P. , Rahmann, S. , Metz, C. , Bornfeld, N. , … Zeschnigk, M. (2013). Exome sequencing identifies recurrent somatic mutations in EIF1AX and SF3B1 in uveal melanoma with disomy 3. Nature Genetics, 45(8), 933–936. 10.1038/ng.2674 23793026 PMC4307600

[pcmr12853-bib-0095] Matatall, K. A. , Agapova, O. A. , Onken, M. D. , Worley, L. A. , Bowcock, A. M. , & Harbour, J. W. (2013). BAP1 deficiency causes loss of melanocytic cell identity in uveal melanoma. BMC Cancer, 13, 371 10.1186/1471-2407-13-371 23915344 PMC3846494

[pcmr12853-bib-0096] McLaughlin, C. C. , Wu, X. C. , Jemal, A. , Martin, H. J. , Roche, L. M. , & Chen, V. W. (2005). Incidence of noncutaneous melanomas in the U.S. Cancer, 103(5), 1000–1007. 15651058 10.1002/cncr.20866

[pcmr12853-bib-0097] Mercer, K. , Giblett, S. , Green, S. , Lloyd, D. , DaRocha Dias, S. , Plumb, M. , … Pritchard, C. (2005). Expression of endogenous oncogenic V600EB‐raf induces proliferation and developmental defects in mice and transformation of primary fibroblasts. Cancer Research, 65(24), 11493–11500. 16357158 10.1158/0008-5472.CAN-05-2211 PMC2640458

[pcmr12853-bib-0098] Meyers, J. R. (2018). Zebrafish: Development of a vertebrate model organism. Current Protocols Essential Laboratory Techniques, 16, e19.

[pcmr12853-bib-0099] Moore, A. R. , Ceraudo, E. , Sher, J. J. , Guan, Y. , Shoushtari, A. N. , Chang, M. T. , … Chen, Y. U. (2016). Recurrent activating mutations of G‐protein‐coupled receptor CYSLTR2 in uveal melanoma. Nature Genetics, 48(6), 675–680. 10.1038/ng.3549 27089179 PMC5032652

[pcmr12853-bib-0100] Moore, A. R. , Ran, L. , Guan, Y. , Sher, J. J. , Hitchman, T. D. , Zhang, J. Q. , … Chen, Y. U. (2018). GNA11 Q209L mouse model reveals RasGRP3 as an essential signaling node in uveal melanoma. Cell Reports, 22(9), 2455–2468. 10.1016/j.celrep.2018.01.081 29490280 PMC5854482

[pcmr12853-bib-0101] Mouriaux, F. , Zaniolo, K. , Bergeron, M.‐A. , Weidmann, C. , De La Fouchardière, A. , Fournier, F. , … Guérin, S. L. (2016). Effects of long‐term serial passaging on the characteristics and properties of cell lines derived from uveal melanoma primary tumors. Investigative Ophthalmology & Visual Science, 57(13), 5288–5301. 10.1167/iovs.16-19317 27723895

[pcmr12853-bib-0102] Mouti, M. A. , Dee, C. , Coupland, S. E. , & Hurlstone, A. F. (2016). Minimal contribution of ERK1/2‐MAPK signalling towards the maintenance of oncogenic GNAQQ209P‐driven uveal melanomas in zebrafish. Oncotarget, 7(26), 39654–39670. 27166257 10.18632/oncotarget.9207 PMC5129960

[pcmr12853-bib-0103] Musi, E. , Ambrosini, G. , de Stanchina, E. , & Schwartz, G. K. (2014). The phosphoinositide 3‐kinase α selective inhibitor BYL719 enhances the effect of the protein kinase C inhibitor AEB071 in GNAQ/GNA11‐mutant uveal melanoma cells. Molecular Cancer Therapeutics, 13(5), 1044–1053. 10.1158/1535-7163.MCT-13-0550 24563540 PMC4146424

[pcmr12853-bib-0104] National Comprehensive Cancer Network . Uveal Melanoma (Version 1.2019). Retrieved from https://www.nccn.org/professionals/physician_gls/pdf/uveal.pdf 10.6004/jnccn.2020.0007 32023525

[pcmr12853-bib-0105] Neal, J. T. , Li, X. , Zhu, J. , Giangarra, V. , Grzeskowiak, C. L. , Ju, J. , … Kuo, C. J. (2018). Organoid modeling of the tumor immune microenvironment. Cell, 175(7), 1972–1988 e1916. 10.1016/j.cell.2018.11.021 30550791 PMC6656687

[pcmr12853-bib-0106] Némati, F. , de Montrion, C. , Lang, G. , Kraus‐Berthier, L. , Carita, G. , Sastre‐Garau, X. , … Decaudin, D. (2014). Targeting Bcl‐2/Bcl‐XL induces antitumor activity in uveal melanoma patient‐derived xenografts. PLoS ONE, 9(1), e80836 10.1371/journal.pone.0080836 24454684 PMC3890263

[pcmr12853-bib-0107] Nemati, F. , Sastre‐Garau, X. , Laurent, C. , Couturier, J. , Mariani, P. , Desjardins, L. , … Decaudin, D. (2010). Establishment and characterization of a panel of human uveal melanoma xenografts derived from primary and/or metastatic tumors. Clinical Cancer Research, 16(8), 2352–2362. 10.1158/1078-0432.CCR-09-3066 20371695

[pcmr12853-bib-0108] Niederkorn, J. Y. (1984). Enucleation in consort with immunologic impairment promotes metastasis of intraocular melanomas in mice. Investigative Ophthalmology & Visual Science, 25(9), 1080–1086. 6381375

[pcmr12853-bib-0109] Niederkorn, J. Y. (2012). Ocular immune privilege and ocular melanoma: Parallel universes or immunological plagiarism? Frontiers in Immunology, 3, 148 10.3389/fimmu.2012.00148 22707951 PMC3374415

[pcmr12853-bib-0110] Niederkorn, J. Y. , Mellon, J. , Pidherney, M. , Mayhew, E. , & Anand, R. (1993). Effect of anti‐ganglioside antibodies on the metastatic spread of intraocular melanomas in a nude mouse model of human uveal melanoma. Current Eye Research, 12(4), 347–358. 10.3109/02713689308999459 8319494

[pcmr12853-bib-0111] Niederkorn, J. Y. , Sanborn, G. E. , & Gamel, J. W. (1987). Suicide enzyme inhibition as a chemotherapeutic strategy for controlling metastases derived from intraocular melanomas. Investigative Ophthalmology & Visual Science, 28(11), 1844–1850. 3117718

[pcmr12853-bib-0112] Ozaki, S. , Vuyyuru, R. , Kageyama, K. , Terai, M. , Ohara, M. , Cheng, H. , … Sato, T. (2016). Establishment and characterization of orthotopic mouse models for human uveal melanoma hepatic colonization. American Journal of Pathology, 186(1), 43–56. 10.1016/j.ajpath.2015.09.011 26613897 PMC4715216

[pcmr12853-bib-0113] Perez, D. E. , Henle, A. M. , Amsterdam, A. , Hagen, H. R. , & Lees, J. A. (2018). Uveal melanoma driver mutations in GNAQ/11 yield numerous changes in melanocyte biology. Pigment Cell Melanoma Res, 31(5), 604–613. 29570931 10.1111/pcmr.12700 PMC6151293

[pcmr12853-bib-0114] Piquet, L. , Dewit, L. , Schoonjans, N. , Millet, M. , Bérubé, J. , Gerges, P. R. A. , … Landreville, S. (2019). Synergic interactions between hepatic stellate cells and uveal melanoma in metastatic growth. Cancers (Basel), 11(8), 1043 10.3390/cancers11081043 PMC6721369 31344830

[pcmr12853-bib-0115] Poeschinger, T. , Renner, A. , Weber, T. , & Scheuer, W. (2013). Bioluminescence imaging correlates with tumor serum marker, organ weights, histology, and human DNA levels during treatment of orthotopic tumor xenografts with antibodies. Molecular Imaging and Biology, 15(1), 28–39. 10.1007/s11307-012-0559-x 22528864

[pcmr12853-bib-0116] Rajaii, F. , Asnaghi, L. , Enke, R. , Merbs, S. L. , Handa, J. T. , & Eberhart, C. G. (2014). The demethylating agent 5-Aza reduces the growth, invasiveness, and clonogenicity of uveal and cutaneous melanoma. Invest Ophthalmol Vis Sci, 55(10), 6178–6186. 25146981 10.1167/iovs.14-13933

[pcmr12853-bib-0117] Repp, A. C. , Mayhew, E. S. , Howard, K. , Alizadeh, H. , & Niederkorn, J. Y. (2001). Role of fas ligand in uveal melanoma‐induced liver damage. Graefes Archive for Clinical and Experimental Ophthalmology, 239(10), 752–758. 10.1007/s004170100363 11760036

[pcmr12853-bib-0118] Rietschel, P. , Panageas, K. S. , Hanlon, C. , Patel, A. , Abramson, D. H. , & Chapman, P. B. (2005). Variates of survival in metastatic uveal melanoma. Journal of Clinical Oncology, 23(31), 8076–8080. 10.1200/JCO.2005.02.6534 16258106

[pcmr12853-bib-0119] Robertson, A. G. , Shih, J. , Yau, C. , Gibb, E. A. , Oba, J. , Mungall, K. L. , … Zmuda, E. (2017). Integrative analysis identifies four molecular and clinical subsets in uveal melanoma. Cancer Cell, 32(2), 204–220 e15. 10.1016/j.ccell.2017.07.003 28810145 PMC5619925

[pcmr12853-bib-0120] Rusciano, D. , Lorenzoni, P. , & Burger, M. (1994). Murine models of liver metastasis. Invasion and Metastasis, 14(1–6), 349–361. 7657528

[pcmr12853-bib-0121] Sachs, N. , de Ligt, J. , Kopper, O. , Gogola, E. , Bounova, G. , Weeber, F. , … Clevers, H. (2018). A living biobank of breast cancer organoids captures disease heterogeneity. Cell, 172(1–2), 373–386 e10. 10.1016/j.cell.2017.11.010 29224780

[pcmr12853-bib-0122] Samadi, A. K. , Cohen, S. M. , Mukerji, R. , Chaguturu, V. , Zhang, X. , Timmermann, B. N. , … Person, E. A. (2012). Natural withanolide withaferin A induces apoptosis in uveal melanoma cells by suppression of Akt and c‐MET activation. Tumour Biology, 33(4), 1179–1189. 10.1007/s13277-012-0363-x 22477711

[pcmr12853-bib-0123] Sanborn, G. E. , Niederkorn, J. Y. , & Gamel, J. W. (1992). Efficacy of dacarbazine (DTIC) in preventing metastases arising from intraocular melanomas in mice. Graefes Archive for Clinical and Experimental Ophthalmology, 230(2), 192–196. 10.1007/BF00164663 1577304

[pcmr12853-bib-0124] Sanborn, G. , Niederkorn, J. , Kan‐Mitchell, J. , & Albert, D. (1992). Prevention of metastasis of intraocular melanoma in mice treated with difluoromethylornithine. Graefes Archive for Clinical and Experimental Ophthalmology, 230(1), 72–77. 10.1007/BF00166766 1547972

[pcmr12853-bib-0125] Schiffner, S. , Braunger, B. M. , de Jel, M. M. , Coupland, S. E. , Tamm, E. R. , & Bosserhoff, A. K. (2014). Tg(Grm1) transgenic mice: A murine model that mimics spontaneous uveal melanoma in humans? Experimental Eye Research, 127, 59–68. 10.1016/j.exer.2014.07.009 25051141

[pcmr12853-bib-0126] Schrage, R. , Schmitz, A.‐L. , Gaffal, E. , Annala, S. , Kehraus, S. , Wenzel, D. , … Kostenis, E. (2015). The experimental power of FR900359 to study Gq‐regulated biological processes. Nature Communications, 6, 10156 10.1038/ncomms10156 PMC4682109 26658454

[pcmr12853-bib-0127] Shain, A. H. , Bagger, M. M. , Yu, R. , Chang, D. , Liu, S. , Vemula, S. , … Kiilgaard, J. F. (2019). The genetic evolution of metastatic uveal melanoma. Nature Genetics, 51(7), 1123–1130. 10.1038/s41588-019-0440-9 31253977 PMC6632071

[pcmr12853-bib-0128] Siolas, D. , & Hannon, G. J. (2013). Patient‐derived tumor xenografts: Transforming clinical samples into mouse models. Cancer Research, 73(17), 5315–5319. 10.1158/0008-5472.CAN-13-1069 23733750 PMC3766500

[pcmr12853-bib-0129] Stei, M. M. , Loeffler, K. U. , Holz, F. G. , & Herwig, M. C. (2016). Animal models of uveal melanoma: Methods, applicability, and limitations. BioMed Research International, 2016, 4521807 10.1155/2016/4521807 27366747 PMC4913058

[pcmr12853-bib-0130] Stei, M. M. , Loeffler, K. U. , Kurts, C. , Hoeller, T. , Pfarrer, C. , Holz, F. G. , & Herwig‐Carl, M. C. (2016). Impact of macrophages on tumor growth characteristics in a murine ocular tumor model. Experimental Eye Research, 151, 9–18. 10.1016/j.exer.2016.07.008 27426931

[pcmr12853-bib-0131] Suesskind, D. , Gauss, S. , Faust, U. E. , Bauer, P. , Schrader, M. , Bartz‐Schmidt, K. U. , & Henke‐Fahle, S. (2013). Characterisation of novel uveal melanoma cell lines under serum‐free conditions. Graefes Archive for Clinical and Experimental Ophthalmology, 251(8), 2063–2070. 10.1007/s00417-013-2292-9 23456173

[pcmr12853-bib-0132] Surriga, O. , Rajasekhar, V. K. , Ambrosini, G. , Dogan, Y. , Huang, R. , & Schwartz, G. K. (2013). Crizotinib, a c‐Met inhibitor, prevents metastasis in a metastatic uveal melanoma model. Molecular Cancer Therapeutics, 12(12), 2817–2826. 10.1158/1535-7163.MCT-13-0499 24140933

[pcmr12853-bib-0133] Süsskind, D. , Hurst, J. , Rohrbach, J. M. , & Schnichels, S. (2017). Novel mouse model for primary uveal melanoma: A pilot study. Clinical & Experimental Ophthalmology, 45(2), 192–200. 10.1111/ceo.12814 27505446

[pcmr12853-bib-0134] Sutmuller, R. P. M. , Schurmans, L. R. H. M. , van Duivenvoorde, L. M. , Tine, J. A. , van der Voort, E. I. H. , Toes, R. E. M. , … Offringa, R. (2000). Adoptive T cell immunotherapy of human uveal melanoma targeting gp100. The Journal of Immunology, 165(12), 7308–7315. 10.4049/jimmunol.165.12.7308 11120866

[pcmr12853-bib-0135] Syed, N. A. , Windle, J. J. , Darjatmoko, S. R. , Lokken, J. M. , Steeves, R. A. , Chappell, R. , … Albert, D. M. (1998). Transgenic mice with pigmented intraocular tumors: Tissue of origin and treatment. Investigative Ophthalmology & Visual Science, 39(13), 2800–2805. 9856795

[pcmr12853-bib-0136] Tafreshi, N. K. , Tichacek, C. J. , Pandya, D. N. , Doligalski, M. L. , Budzevich, M. M. , Kil, H. J. , … Morse, D. L. (2019). Melanocortin 1 receptor‐targeted α‐particle therapy for metastatic uveal melanoma. Journal of Nuclear Medicine, 60(8), 1124–1133. 10.2967/jnumed.118.217240 30733316 PMC6681690

[pcmr12853-bib-0137] Tolleson, W. H. , Doss, J. C. , Latendresse, J. , Warbritton, A. R. , Melchior, W. B. Jr , Chin, L. , … Albert, D. M. (2005). Spontaneous uveal amelanotic melanoma in transgenic Tyr‐RAS+ Ink4a/Arf‐/‐ mice. Archives of Ophthalmology, 123(8), 1088–1094. 10.1001/archopht.123.8.1088 16087843

[pcmr12853-bib-0138] Valyi‐Nagy, T. , Fredericks, B. , Ravindra, A. , Hopkins, J. , Shukla, D. , & Valyi‐Nagy, K. (2018). Herpes simplex virus 1 infection promotes the growth of a subpopulation of tumor cells in three‐dimensional uveal melanoma cultures. Journal of Virology, 92(19), e00700–e718. 10.1128/JVI.00700-18 30045986 PMC6146807

[pcmr12853-bib-0139] van der Ent, W. , Burrello, C. , Teunisse, A. F. A. S. , Ksander, B. R. , van der Velden, P. A. , Jager, M. J. , … Snaar‐Jagalska, B. E. (2014). Modeling of human uveal melanoma in zebrafish xenograft embryos. Investigative Ophthalmology & Visual Science, 55(10), 6612–6622. 10.1167/iovs.14-15202 25249605

[pcmr12853-bib-0140] Van Raamsdonk, C. D. , Bezrookove, V. , Green, G. , Bauer, J. , Gaugler, L. , O'Brien, J. M. , … Bastian, B. C. (2009). Frequent somatic mutations of GNAQ in uveal melanoma and blue naevi. Nature, 457(7229), 599–602. 19078957 10.1038/nature07586 PMC2696133

[pcmr12853-bib-0141] Van Raamsdonk, C. D. , Griewank, K. G. , Crosby, M. B. , Garrido, M. C. , Vemula, S. , Wiesner, T. , … Bastian, B. C. (2010). Mutations in GNA11 in uveal melanoma. New England Journal of Medicine, 363(23), 2191–2199. 21083380 10.1056/NEJMoa1000584 PMC3107972

[pcmr12853-bib-0142] VanBrocklin, M. W. , Robinson, J. P. , Lastwika, K. J. , Khoury, J. D. , & Holmen, S. L. (2010). Targeted delivery of NRASQ61R and Cre‐recombinase to post‐natal melanocytes induces melanoma in Ink4a/Arflox/lox mice. Pigment Cell & Melanoma Research, 23(4), 531–541. 10.1111/j.1755-148X.2010.00717.x 20444198 PMC2906690

[pcmr12853-bib-0143] Vaqué, J. P. , Dorsam, R. T. , Feng, X. , Iglesias‐Bartolome, R. , Forsthoefel, D. J. , Chen, Q. , … Gutkind, J. S. (2013). A genome‐wide RNAi screen reveals a Trio‐regulated Rho GTPase circuitry transducing mitogenic signals initiated by G protein‐coupled receptors. Molecular Cell, 49(1), 94–108. 10.1016/j.molcel.2012.10.018 23177739 PMC3545055

[pcmr12853-bib-0144] Verbik, D. J. , Murray, T. G. , Tran, J. M. , & Ksander, B. R. (1997). Melanomas that develop within the eye inhibit lymphocyte proliferation. International Journal of Cancer, 73(4), 470–478. 10.1002/(SICI)1097-0215(19971114)73:4<470:AID-IJC3>3.0.CO;2-X 9389558

[pcmr12853-bib-0145] Vivet‐Noguer, R. , Tarin, M. , Roman‐Roman, S. , & Alsafadi, S. (2019). Emerging therapeutic opportunities based on current knowledge of uveal melanoma biology. Cancers (Basel), 11(7), 1019 10.3390/cancers11071019 PMC6678734 31330784

[pcmr12853-bib-0146] Vlachogiannis, G. , Hedayat, S. , Vatsiou, A. , Jamin, Y. , Fernandez‐Mateos, J. , Khan, K. , … Valeri, N. (2018). Patient‐derived organoids model treatment response of metastatic gastrointestinal cancers. Science, 359(6378), 920–926. 29472484 10.1126/science.aao2774 PMC6112415

[pcmr12853-bib-0147] Voropaev, H. , Gimmelshein Vatkin, M. , Shneor, D. , Luski, S. , Honigman, A. , & Frenkel, S. (2019). Infectious knockdown of CREB and HIF‐1 for the treatment of metastatic uveal melanoma. Cancers (Basel), 11(8), 1056 10.3390/cancers11081056 PMC6721386 31357444

[pcmr12853-bib-0148] Wang, Y. , Liu, M. , Jin, Y. , Jiang, S. , & Pan, J. (2017). In vitro and in vivo anti‐uveal melanoma activity of JSL‐1, a novel HDAC inhibitor. Cancer Letters, 400, 47–60. 28455241 10.1016/j.canlet.2017.04.028

[pcmr12853-bib-0149] Wege, A. K. (2018). Humanized mouse models for the preclinical assessment of cancer immunotherapy. BioDrugs: Clinical Immunotherapeutics, Biopharmaceuticals and Gene Therapy, 32(3), 245–266. 10.1007/s40259-018-0275-4 29589229

[pcmr12853-bib-0150] Xue, S. , Yang, H. , Qiao, J. , Pu, F. , Jiang, J. , Hubbard, K. , … Yang, J. J. (2015). Protein MRI contrast agent with unprecedented metal selectivity and sensitivity for liver cancer imaging. Proceedings of the National Academy of Sciences of the USA, 112(21), 6607–6612. 10.1073/pnas.1423021112 25971726 PMC4450423

[pcmr12853-bib-0151] Yang, H. , Brackett, C. M. , Morales‐Tirado, V. M. , Li, Z. , Zhang, Q. , Wilson, M. W. , … Grossniklaus, H. E. (2016). The Toll‐like receptor 5 agonist entolimod suppresses hepatic metastases in a murine model of ocular melanoma via an NK cell‐dependent mechanism. Oncotarget, 7(3), 2936–2950. 10.18632/oncotarget.6500 26655090 PMC4823082

[pcmr12853-bib-0152] Yang, H. , Cao, J. , & Grossniklaus, H. E. (2015). Uveal melanoma metastasis models. Ocular Oncology and Pathology, 1(3), 151–160. 10.1159/000370153 27171919 PMC4847657

[pcmr12853-bib-0153] Yang, H. , & Grossniklaus, H. E. (2010). Constitutive overexpression of pigment epithelium‐derived factor inhibition of ocular melanoma growth and metastasis. Investigative Ophthalmology & Visual Science, 51(1), 28–34. 10.1167/iovs.09-4138 19661223 PMC2819011

[pcmr12853-bib-0154] Yang, H. , Jager, M. J. , & Grossniklaus, H. E. (2010). Bevacizumab suppression of establishment of micrometastases in experimental ocular melanoma. Investigative Ophthalmology & Visual Science, 51(6), 2835–2842. 10.1167/iovs.09-4755 20089875 PMC2874122

[pcmr12853-bib-0155] Yang, H. , Sun, L. , Liu, M. , & Mao, Y. (2018). Patient‐derived organoids: A promising model for personalized cancer treatment. Gastroenterology Report (Oxf), 6(4), 243–245. 10.1093/gastro/goy040 PMC6225812 30430011

[pcmr12853-bib-0156] Yang, H. , Xu, Z. , Iuvone, P. M. , & Grossniklaus, H. E. (2006). Angiostatin decreases cell migration and vascular endothelium growth factor (VEGF) to pigment epithelium derived factor (PEDF) RNA ratio in vitro and in a murine ocular melanoma model. Molecular Vision, 12, 511–517. 16735992

[pcmr12853-bib-0157] Yang, J. , Manson, D. K. , Marr, B. P. , & Carvajal, R. D. (2018). Treatment of uveal melanoma: Where are we now? Therapeutic Advances in Medical Oncology, 10, 1758834018757175 10.1177/1758834018757175 29497459 PMC5824910

[pcmr12853-bib-0158] Yang, W. , Li, H. , Mayhew, E. , Mellon, J. , Chen, P. W. , & Niederkorn, J. Y. (2011). NKT cell exacerbation of liver metastases arising from melanomas transplanted into either the eyes or spleens of mice. Investigative Ophthalmology & Visual Science, 52(6), 3094–3102. 10.1167/iovs.10-7067 21330669 PMC3109018

[pcmr12853-bib-0159] Yoo, J. H. , Shi, D. S. , Grossmann, A. H. , Sorensen, L. K. , Tong, Z. Z. , Mleynek, T. M. , … Li, D. Y. (2016). ARF6 is an actionable node that orchestrates oncogenic GNAQ signaling in uveal melanoma. Cancer Cell, 29(6), 889–904. 10.1016/j.ccell.2016.04.015 27265506 PMC5027844

[pcmr12853-bib-0160] Yoshida, M. , Selvan, S. , McCue, P. A. , DeAngelis, T. , Baserga, R. , Fujii, A. , … Sato, T. (2014). Expression of insulin‐like growth factor‐1 receptor in metastatic uveal melanoma and implications for potential autocrine and paracrine tumor cell growth. Pigment Cell & Melanoma Research, 27(2), 297–308. 10.1111/pcmr.12206 24354797

[pcmr12853-bib-0161] Yu, F.‐X. , Luo, J. , Mo, J.‐S. , Liu, G. , Kim, Y. C. , Meng, Z. , … Guan, K.‐L. (2014). Mutant Gq/11 promote uveal melanoma tumorigenesis by activating YAP. Cancer Cell, 25(6), 822–830. 10.1016/j.ccr.2014.04.017 24882516 PMC4075337

[pcmr12853-bib-0162] Yu, X. , Ambrosini, G. , Roszik, J. , Eterovic, A. K. , Stempke‐Hale, K. , Seftor, E. A. , … Woodman, S. E. (2015). Genetic analysis of the 'uveal melanoma' C918 cell line reveals atypical BRAF and common KRAS mutations and single tandem repeat profile identical to the cutaneous melanoma C8161 cell line. Pigment Cell & Melanoma Research, 28(3), 357–359. 25515650 10.1111/pcmr.12345

[pcmr12853-bib-0163] Zhang, Q. , Yang, H. , Kang, S. J. , Wang, Y. , Wang, G. D. , Coulthard, T. , & Grossniklaus, H. E. (2011). In vivo high‐frequency, contrast‐enhanced ultrasonography of uveal melanoma in mice: Imaging features and histopathologic correlations. Investigative Ophthalmology & Visual Science, 52(5), 2662–2668. 10.1167/iovs.10-6794 21245408 PMC3088556

[pcmr12853-bib-0164] Zhou, J. , Jin, B. , Jin, Y. , Liu, Y. , & Pan, J. (2017). The antihelminthic drug niclosamide effectively inhibits the malignant phenotypes of uveal melanoma in vitro and in vivo. Theranostics, 7(6), 1447–1462. 28529629 10.7150/thno.17451 PMC5436505

[pcmr12853-bib-0165] Zhu, A. , Zhan, W. , Liang, Z. , Yoon, Y. , Yang, H. , Grossniklaus, H. E. , … Shim, H. (2010). Dipyrimidine amines: A novel class of chemokine receptor type 4 antagonists with high specificity. Journal of Medicinal Chemistry, 53(24), 8556–8568. 10.1021/jm100786g 21105715 PMC3003753

[pcmr12853-bib-0166] Zitvogel, L. , Pitt, J. M. , Daillere, R. , Smyth, M. J. , & Kroemer, G. (2016). Mouse models in oncoimmunology. Nature Reviews Cancer, 16(12), 759–773. 10.1038/nrc.2016.91 27687979

[pcmr12853-bib-0167] Zuberi, A. , & Lutz, C. (2016). Mouse models for drug discovery. Can new tools and technology improve translational power? ILAR Journal, 57(2), 178–185. 28053071 10.1093/ilar/ilw021 PMC5886322

